# Enhanced tyrosine sulfation is associated with chronic kidney disease-related atherosclerosis

**DOI:** 10.1186/s12915-023-01641-y

**Published:** 2023-07-10

**Authors:** Daopeng Dai, Zhengbin Zhu, Hui Han, Tian Xu, Shuo Feng, Wenli Zhang, Fenghua Ding, Ruiyan Zhang, Jinzhou Zhu

**Affiliations:** 1 Department of Vascular & Cardiology, Ruijin Hospital, Shanghai Jiao Tong University School of Medicine, 197 Ruijin Road II, Shanghai, 200025 China; 2 Institute of Cardiovascular Diseases, Shanghai Jiao Tong University School of Medicine, Shanghai, China; 3 Department of Nephrology, Ruijin Hospital, Shanghai Jiao Tong University School of Medicine, Shanghai, China

**Keywords:** Chronic kidney disease, Tyrosine sulfation, Atherosclerosis

## Abstract

**Background:**

Chronic kidney disease (CKD) accelerates atherosclerosis, but the mechanisms remain unclear. Tyrosine sulfation has been recognized as a key post-translational modification (PTM) in regulation of various cellular processes, and the sulfated adhesion molecules and chemokine receptors have been shown to participate in the pathogenesis of atherosclerosis via enhancement of monocyte/macrophage function. The levels of inorganic sulfate, the essential substrate for the sulfation reaction, are dramatically increased in patients with CKD, which indicates a change of sulfation status in CKD patients. Thus, in the present study, we detected the sulfation status in CKD patients and probed into the impact of sulfation on CKD-related atherosclerosis by targeting tyrosine sulfation function.

**Results:**

PBMCs from individuals with CKD showed higher amounts of total sulfotyrosine and tyrosylprotein sulfotransferase (TPST) type 1 and 2 protein levels. The plasma level of O-sulfotyrosine, the metabolic end product of tyrosine sulfation, increased significantly in CKD patients. Statistically, O-sulfotyrosine and the coronary atherosclerosis severity SYNTAX score positively correlated. Mechanically, more sulfate-positive nucleated cells in peripheral blood and more abundant infiltration of sulfated macrophages in deteriorated vascular plaques in CKD ApoE null mice were noted. Knockout of TPST1 and TPST2 decreased atherosclerosis and peritoneal macrophage adherence and migration in CKD condition. The sulfation of the chemokine receptors, CCR2 and CCR5, was increased in PBMCs from CKD patients.

**Conclusions:**

CKD is associated with increased sulfation status. Increased sulfation contributes to monocyte/macrophage activation and might be involved in CKD-related atherosclerosis. Inhibition of sulfation may suppress CKD-related atherosclerosis and is worthy of further study.

**Supplementary Information:**

The online version contains supplementary material available at 10.1186/s12915-023-01641-y.

## Background

Chronic kidney disease (CKD) accelerates cardiovascular disease (CVD) [1], and coronary artery atherosclerosis is highly prevalent and thought to be a major cause of mortality in CKD patients [2, 3]. CKD-related hypertension, dyslipidemia, and diabetes likely accelerate coronary atherosclerosis but cannot fully explain the frequency and severity of atherosclerosis, as the Framingham risk score based on these traditional risk factors provides poor overall accuracy in predicting cardiovascular events in CKD population [4, 5]. Nontraditional risk factors, including oxidative stress, inflammation, renin-angiotensin signaling, volume overload, and calcium and phosphorus metabolism disorders, may also contribute to the progression of CKD-related atherosclerosis [6–8]. Our previous study also revealed that one protein-bound uremic toxin retained under CKD conditions, p-cresyl sulfate (PCS), could promote uremic atherosclerosis [9]. Nevertheless, the mechanisms responsible for CKD-specific atherosclerosis are still not completely known.

Tyrosine sulfation is a common post-translational modification (PTM) of the cell membrane and secreted proteins that occurs in the trans-Golgi network [10]. The modification transfers sulfate from 3’-phosphate 5’-phosphosulfate (PAPS) to tyrosine residues of target proteins, which is catalyzed by tyrosylprotein sulfotransferases (TPST) 1 and 2 [11]. Tyrosine sulfation plays an essential role in regulating protein–protein interactions and is implicated in several biological processes such as hemostasis, inflammation response, and chemokine receptor recognition [12]. Of relevance, sulfated proteins include several chemokine receptors (e.g., CCR2, CCR5, CX3CR1) and adhesion molecules (e.g., PSGL1), which are implicated in atherosclerosis [13–17]. In fact, it has been reported that excessive tyrosine sulfation of leukocyte adhesion molecules and chemokine receptors promotes atherosclerosis [18], and deletion of TPST activity in myeloid cells attenuates atherosclerosis in LDLR^−/−^ mice [19].

Sulfation status in CKD has not been studied. However, studies have found that inorganic sulfate, which is the essential substrate for sulfation of tyrosine residues, dramatically increases in CKD patients, implicating a change of sulfation level in CKD condition [20]. In fact, our previous study found that O-sulfotyrosine, the metabolite of tyrosine sulfation, increases significantly under CKD condition [21]. Herein, we tested the hypothesis that tyrosine sulfation is increased in CKD patients and plays an important role in the development of CKD-related atherosclerosis.

## Results

### The increased sulfation status under CKD condition

**Fig. 1:**
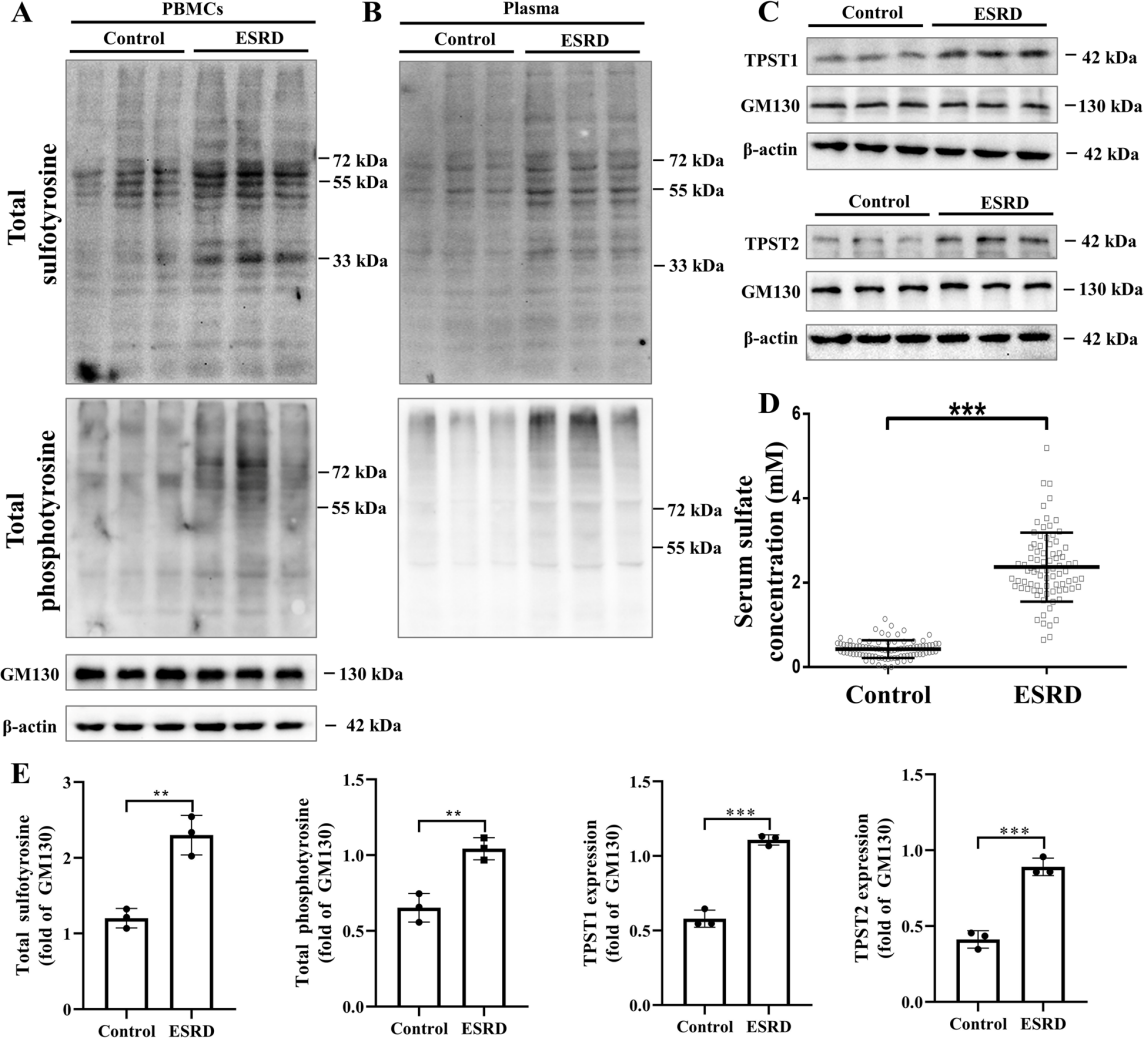
Increased tyrosine sulfation in CKD patients. **A** Western blot analyses showed increased total sulfotyrosine level in PBMCs extracted from ESRD patients compared with control subjects. Total phosphotyrosine level was also increased in PBMCs from ESRD patients. **B** Total sulfotyrosine and total phosphotyrosine levels were higher in plasma from ESRD patients compared with control subjects. **C** Western blot analyses showed increased TPST1 and TPST2 protein levels in PBMCs extracted from ESRD patients compared with control subjects. **D** Plasma sulfate levels increased significantly in ESRD patients (*n* = 81) compared with controls (*n* = 90). Band densitometry of total sulfotyrosine, total phosphotyrosine, TPST1, and TPST2 was quantified in E. PBMCs samples from six patients in the same group were mixed in one lane for western blot analysis. Data are expressed as means ± SD. ^**^p < 0.01 compared with the control group, ^***^p < 0.001 compared with the control group. ESRD, end-stage renal disease; PBMC, peripheral blood mononuclear cell; TPST, tyrosyl-protein sulfotransferase

To explore the sulfation status in CKD patients, blood samples were collected and peripheral blood mononuclear cells (PBMCs) and plasma were purified from end stage renal disease (ESRD) patients and controls. Western blot revealed a higher level of total sulfotyrosine in both PBMCs and plasma in ESRD patients compared with controls (Fig. 1A + B). The expression of TPST1 and TPST2, the two main tyrosylprotein sulfotransferases, was higher in PBMCs from ESRD patients (Fig. 1C). A dramatically higher plasma sulfate level in patients with ESRD (2.37 ± 0.82 mM) than in controls (0.43 ± 0.21 mM, *p* < 0.001) was confirmed (Fig. 1D). Interestingly, total phosphotyrosine level was also increased in PBMCs and plasma from ESRD patients, indicating the complex pathophysiological state of kidney dysfunction. Overall, these results indicate the increased sulfation status under CKD condition. Clinical characteristics of the enrolled ESRD and control subjects are shown in Additional File 1: Table S1.

The clinical finding of increased sulfation under CKD condition was further confirmed by animal studies. C57BL/6 mice subjected to 5/6 nephrectomy showed time-dependent increase in tyrosine sulfation, as western blot analysis revealed that bone marrow total sulfotyrosine, TPST1, and TPST2 expression levels gradually increased after 1, 2, 4, 8, and 16 weeks of 5/6 nephrectomy (Additional File 2: Fig. S1). Immunohistochemical staining confirmed that total sulfotyrosine, TPST1, and TPST2 expression levels increased significantly in bone marrow tissues after 16 weeks of 5/6 nephrectomy (Additional File 3: Fig. S2).

### O-sulfotyrosine, the end product of tyrosine sulfation, is significantly associated with coronary atherosclerosis severity SYNTAX score in CKD patients

**Fig. 2:**
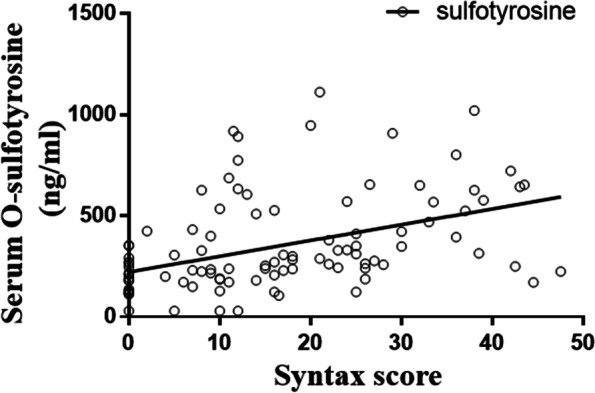
Clinical relevance between tyrosine sulfation and atherosclerosis in CKD patients. Pearson’s correlation coefficients showed that concentration of O-sulfotyrosine, the metabolic end product of tyrosine sulfation, positively correlated with SYNTAX score in individuals with CKD. r = 0.4372, R^2^ = 0.1912, *p* < 0.001, Y = 7.808 × X + 221.8. CKD, chronic kidney disease

Plasma O-sulfotyrosine, the end product of tyrosine sulfation, has been shown to increase in CKD patients in our previous work [21]. Here, plasma O-sulfotyrosine level was detected in CKD patients (cohort 2) to explore its associations with the severity of coronary atherosclerosis assessed by SYNTAX score. Bivariate correlation models for plasma O-sulfotyrosine are shown in Table 1. In unadjusted analysis, O-sulfotyrosine levels and SYNTAX score positively correlated (r = 0.4372, R^2^ = 0.1912, *p* < 0.001) (Fig. 2). There were no significant differences in plasma O-sulfotyrosine levels between males, alcohol users, smokers, or patients with hypertension and their counterparts (Student’s *t* test, *p* > 0.05, data not shown). As expected, O-sulfotyrosine levels and sulfate concentrations positively correlated (r = 0.6921, R^2^ = 0.4790, *p* < 0.001) (Additional File 4: Fig. S3).

**Table 1:** Bivariate correlation models for plasma O-sulfotyrosine in the study cohort 2

	OR	*P* value
Age (years)	− 0.104	0.285
BMI (kg/m^2^)	− 0.169	0.083
WBC (10^9^/L)	− 0.033	0.739
Neutrophil (%)	0.206	0.034
BUN (mmol/L)	0.476	0.000
CRE (μmol/L)	0.635	0.000
UA (μmol/L)	− 0.027	0.784
CysC (mg/L)	0.665	0.000
eGFR (mL/min/1.73 m^2^)	− 0.632	0.000
Fast glucose (mmol/L)	0.016	0.867
HbA1c (%)	− 0.137	0.169
TC (mmol/L)	0.047	0.635
TG (mmol/L)	− 0.071	0.468
HDL-C (mmol/L)	− 0.018	0.854
LDL-C (mmol/L)	0.094	0.337
hsCRP (mg/L)	0.202	0.038

*BMI* Body mass index, *WBC* White blood cells, *BUN* Blood urea nitrogen, *CRE* Creatinine, *UA* Uric acid, *CysC* Cystatin C, *GFR* Glomerular filtration rate, *TC* Total cholesterol, *TG* Triglycerides, *HDL-C* High-density lipoprotein cholesterol, *LDL-C* Low-density lipoprotein cholesterol, *hsCRP* High-sensitivity C-reactive protein

Next, patients were divided into two groups according to SYNTAX score. Table 2 depicts the clinical and demographic characteristics of the angiography cohort that underwent SYNTAX scoring. There were significant differences in O-sulfotyrosine between the high-SYNTAX-score (> 22) and the low-SYNTAX-score (≤ 22) groups. The results of univariate binary logistic regression and multivariate logistic analyses to correlate the coronary atherosclerosis severity SYNTAX score in CKD patients are listed in Table 3, showing that O-sulfotyrosine level was an independent predictor for the high SYNTAX-score (> 22). These results revealed the associations between tyrosine sulfation and CKD-related atherosclerosis.

**Table 2:** Baseline characteristics of CKD patients according to Syntax Score in the study cohort 2

	**SS ≤ 22 (*n* = 73)**	**SS > 22 (*n* = 34)**	***P* value**
Male (%)	49 (67.1)	23 (67.6)	0.957
Age (years)	69.2 (10.97)	69.7 (9.57)	0.835
BMI (kg/m^2^)	24.2 (3.58)	24.4 (3.50)	0.696
Alcohol users (%)	8 (11.0)	6 (17.6)	0.339
Cigarette smoking (%)	7 (9.6)	5 (14.7)	0.435
Hypertension (%)	58 (79.5)	32 (94.1)	0.053
WBC (10^9^/L)	5.95 (1.46)	5.89 (1.36)	0.857
Neutrophil (%)	62.54 (9.04)	65.25 (10.39)	0.171
BUN (mmol/L)	12.04 (6.69)	13.36 (8.59)	0.388
CRE (μmol/L)	247.52 (283.44)	282.74 (249.18)	0.536
UA (μmol/L)	438.26 (107.56)	436.59 (91.93)	0.938
CysC (mg/L)	2.45 (1.97)	2.98 (1.95)	0.201
eGFR (mL/min/1.73 m^2^)	36.9 (16.93)	30.6 (19.26)	0.091
TC (mmol/L)	4.07 (1.26)	3.91 (1.01)	0.498
TG (mmol/L)	1.70 (1.67)	1.41 (0.58)	0.339
HDL-C (mmol/L)	1.08 (0.28)	1.04 (0.23)	0.515
LDL-C (mmol/L)	2.41 (0.92)	2.37 (0.92)	0.848
hsCRP (mg/L)	5.53 (12.31)	10.22 (19.69)	0.139
Plasma O-sulfotyrosine (ng/mL)	294.5 (228.41)	453.3 (221.38)	0.001
Sulfate (mmol/L)	1.87 (0.79)	2.40 (0.80)	0.002

Results are expressed as the mean ± SD. SS, SYNTAX score.

*BMI* Body mass index, *WBC* White blood cells, *BUN* Blood urea nitrogen, *CRE* Creatinine, *UA* Uric acid, *CysC* Cystatin C, *GFR* Glomerular filtration rate, *TC* Total cholesterol, *TG* Triglycerides, *HDL-C* High-density lipoprotein-cholesterol, *LDL-C* Low-density lipoprotein-cholesterol, *hsCRP* High-sensitivity C-reactive protein.

**Table 3:** Univariate and multivariate binary logistic regression analyses for SYNTAX scores > 22

	Univariate		Multivariable	
	OR	*P* value	OR	*P* value
Plasma O-sulfotyrosine /10	1.029 (1.010–1.048)	0.002	1.028 (1.010–1.047)	0.003
WBC (10^9^/L)	0.974 (0.730–1.299)	0.856	-	
Neutrophil (%)	1.031 (0.987–1.078)	0.172	-	
BUN (mmol/L)	1.024 (0.970–1.081)	0.387	-	
CRE (μmol/L)	1.000 (0.999–1.002)	0.533	-	
UA (μmol/L)	1.000 (0.996–1.004)	0.937	-	
CysC (mg/L)	1.139 (0.931–1.393)	0.205	-	
eGFR (mL/min/1.73 m^2^)	0.981 (0.958–1.003)	0.093	-	
TC (mmol/L)	0.883 (0.617–1.263)	0.359	-	
TG (mmol/L)	0.793 (0.483–1.302)	0.495	-	
HDL-C (mmol/L)	0.588 (0.121–2.867)	0.511	-	
LDL-C (mmol/L)	0.956 (0.610–1.500)	0.846	-	
hsCRP (mg/L)	1.019 (0.992–1.047)	0.163	-	

*WBC *White blood cells, *BUN* Blood urea nitrogen, *CRE* Creatinine, *UA* Uric acid, *CysC* Cystatin C, *GFR* Glomerular filtration rate, *TC* Total cholesterol, *TG* Triglycerides, *HDL-C* High-density lipoprotein cholesterol, *LDL-C* Low-density lipoprotein cholesterol, *hsCRP* High-sensitivity C-reactive protein; —, not in the equation.

### The increased sulfated monocytes/macrophages in CKD-related atherosclerosis

**Fig. 3:**
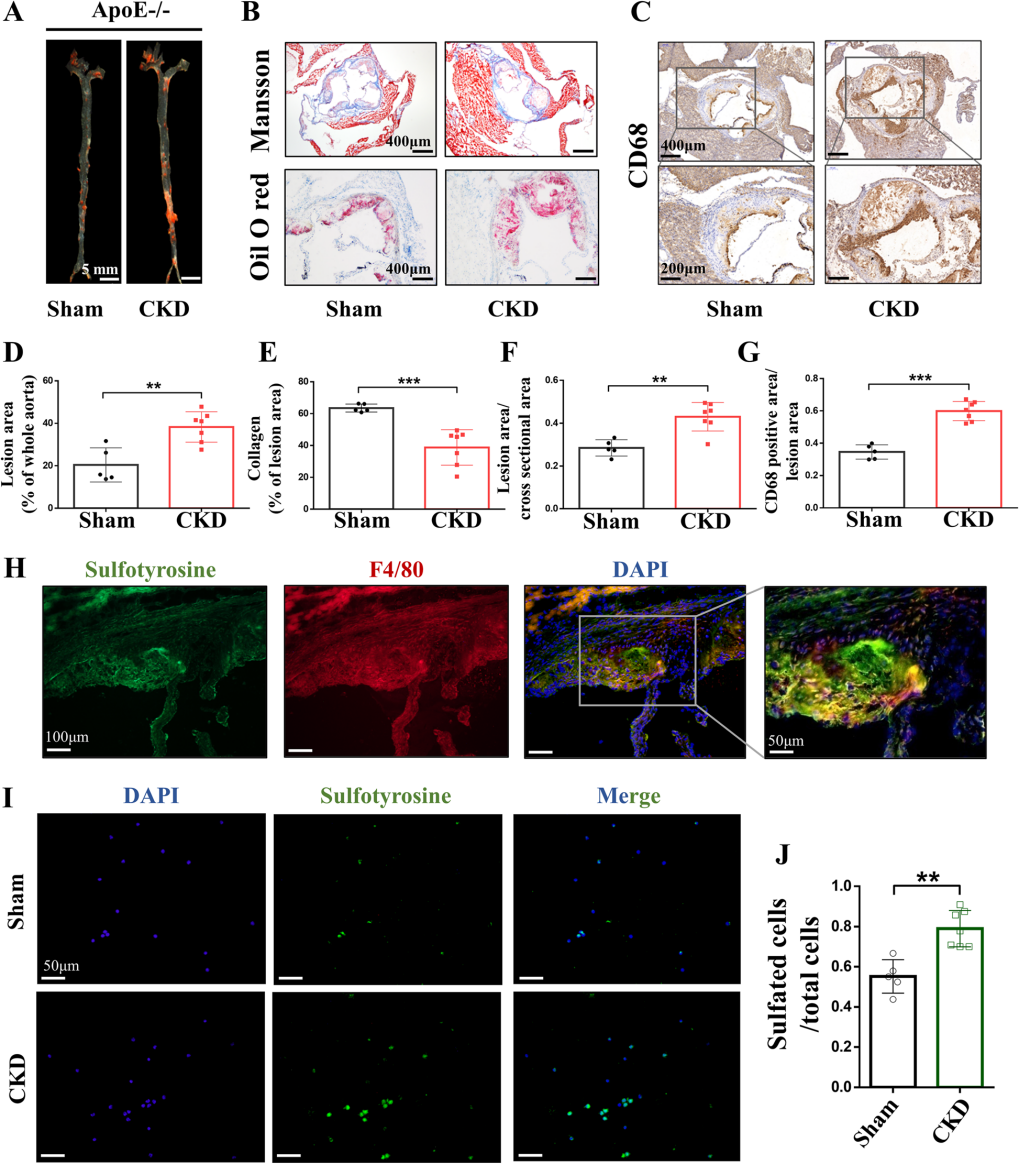
Increased sulfated monocytes/macrophages in CKD-related atherosclerosis. (**A** + **D**) Oil red O staining and quantification of lesions in the aorta en-face showed that atherosclerotic lesion size increased significantly in CKD-ApoE^−/−^ mice compared with Sham-ApoE^−/−^ mice. Scale bar: 2 mm. (**B**) Oil red O staining and Masson-trichrome (collagen in blue) staining of aortic sinus atherosclerotic lesions from CKD-ApoE^−/−^ and Sham-ApoE^−/−^ mice. The results are quantified in **E** + **F**, showing that CKD-ApoE^−/−^ mice have increased aortic sinus atherosclerotic lesions area but lower collagen area. Scale bar: 400 μm in the top image and 200 μm in the lower image. (**C** + **G**) Macrophages highlighted by immunohistochemical staining for CD68 increased significantly in aortic sinus plaques of CKD-ApoE^−/−^ mice compared with Sham-ApoE^−/−^ mice. Scale bar: 400 μm in the top image and 200 μm in the lower image. (**H**) Immunostaining showed co-localization of sulfotyrosine (green) and F4/80 (red) in aortic root sections, indicating the sulfated monocytes/macrophages in CKD-related atherosclerosis. Scale bar: 100 μm in the left image and 50 μm in the right image. (**I** + **J**) Peripheral blood was extracted from Sham-ApoE^−/−^ and CKD-ApoE^−/−^ mice and sulfated nucleated cells were determined by immunofluorescence staining with anti-sulfotyrosine antibody. The results showed that the percentage of sulfotyrosine-positive nucleated cells was significantly increased in CKD-ApoE^−/−^ mice. Scale bar: 50 μm. *n* = 5 for Sham-ApoE^−/−^, *n* = 7 for CKD-ApoE^−/−^. Data are expressed as means ± SD. ^**^p < 0.01, ^***^p < 0.001 compared with the Sham-ApoE^−/−^ group

After demonstrating the increased sulfation status under CKD condition and the positive correlations between the tyrosine sulfation metabolic end product, O-sulfotyrosine, and coronary atherosclerosis severity SYNTAX score, the direct effect of tyrosine sulfation on CKD-related atherosclerosis was further studied. 5/6 nephrectomy and sham-operated ApoE^−/−^ mice were established. In consistence with previous studies [22, 23], atherosclerotic lesion size increased significantly in the CKD group compared with controls, following 12 weeks of a high-cholesterol diet (Fig. 3A + B). CD68 staining, a marker of monocyte/macrophage, was increased in the aortic vascular plaques of CKD-ApoE^−/−^ mice (Fig. 3C). Importantly, immunostaining showed co-localization of sulfotyrosine and F4/80 + monocytes/macrophages in the plaques of CKD-ApoE^−/−^ mice (Fig. 3H). Moreover, the percentage of sulfotyrosine-positive nucleated cells was significantly increased in peripheral blood from CKD-ApoE^−/−^ mice (Fig. 3I). These results indicate that CKD-related atherosclerosis is associated with more abundant infiltration of sulfated monocytes/macrophages. Plasma sulfate levels in ApoE^−/−^ mice at different time points after 5/6 nephrectomy are shown in Additional File 5: Fig. S4.

### Sulfation inhibition attenuates CKD-related atherosclerosis

**Fig. 4:**
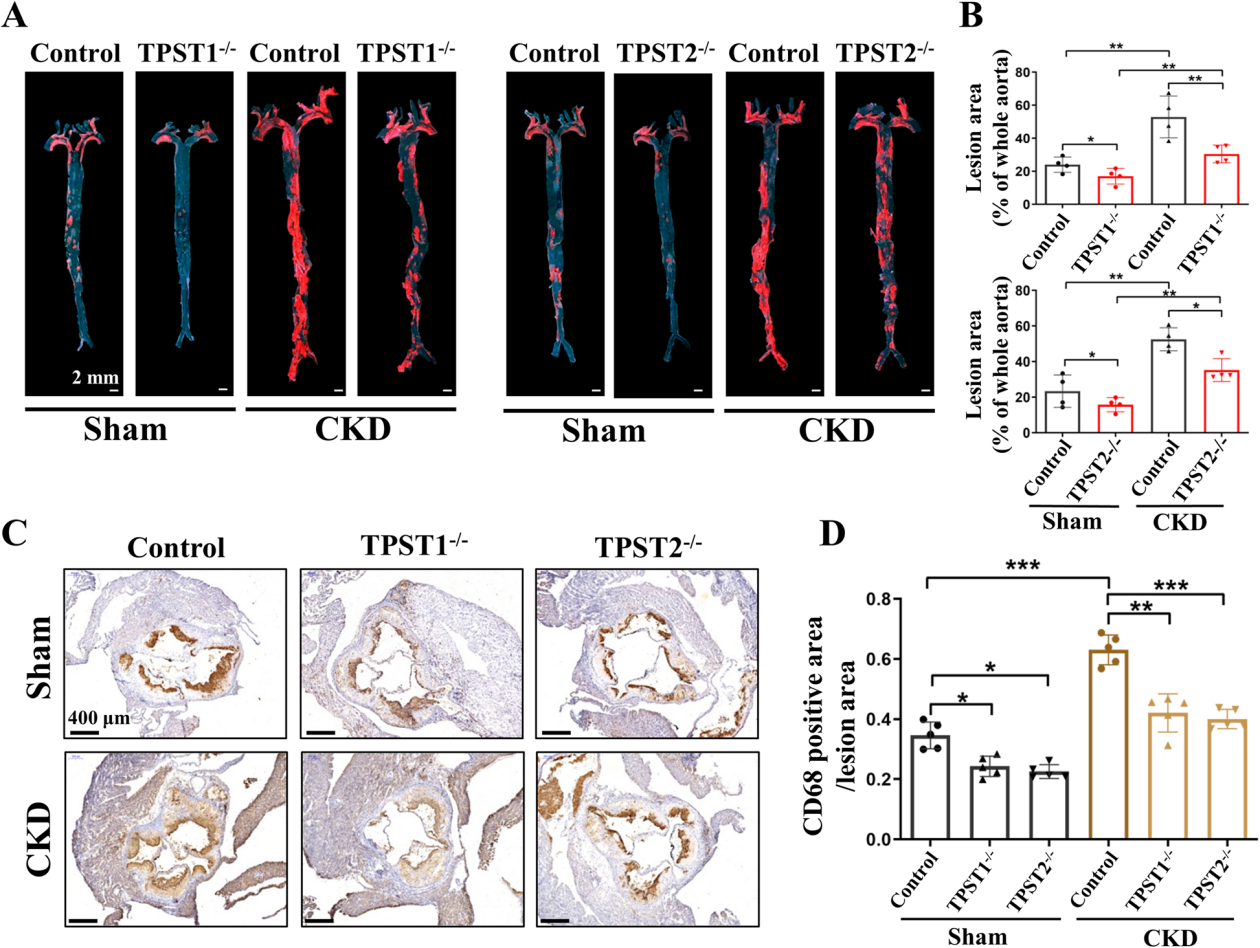
Sulfation inhibition attenuates CKD-related atherosclerosis and decreases the infiltration of monocytes/macrophages. (**A** + **B**) Representative images and analysis of en-face staining with oil red O in aortas from sham and 5/6 nephrectomy ApoE^−/−^ mice with or without TPST knockout. Red staining indicates atherosclerotic plaques. ApoE^−/−^ with TPST1 knockout (TPST1^−/−^) and ApoE^−/−^ with TPST2 knockout (TPST2^−/−^) mice showed smaller atherosclerotic lesion size compared with ApoE^−/−^ (Control) mice. After 5/6 nephrectomy (CKD), ApoE^−/−^ mice presented larger atherosclerotic lesions, while this effect was significantly inhibited in TPST1^−/−^ and TPST2^−/−^ groups. Scale bar: 2 mm, *n* = 4 in each group. (**C** + **D**) The infiltration of CD68 + macrophages in the aortic vascular plaques was alleviated in TPST1^−/−^/ApoE^−/−^ and TPST2^−/−^/ApoE^−/−^ mice with 5/6 nephrectomy. Scale bar: 400 μm, *n* = 5 in each group. Data are expressed as means ± SD. ^*^p < 0.05, ^**^p < 0.01,^***^p < 0.001

TPST1^−/−^ and TPST2^−/−^ mice were first established. We found that both TPST1 and TPST2 knockout were able to decrease tyrosine sulfation (Additional File 6: Fig. S5). TPST1^−/−^/ApoE^−/−^ and TPST2^−/−^/ApoE^−/−^ mice were then established and subjected to 5/6 nephrectomy. Aortic atherosclerotic lesions were analyzed after 12 weeks of a high-cholesterol diet. Notably, both TPST1^−/−^/ApoE^−/−^ and TPST2^−/−^/ApoE^−/−^ mice presented fewer atherosclerotic lesions compared with ApoE^−/−^ mice prior to 5/6 nephrectomy. As expected, 5/6 nephrectomy induced more atherosclerotic lesions in ApoE^−/−^ mice. However, the effect of 5/6 nephrectomy on the lesion size was significantly attenuated in TPST1^−/−^/ApoE^−/−^ and TPST2^−/−^/ApoE^−/−^ mice (Fig. 4A + B). The infiltration of CD68 + monocytes/macrophages in the aortic vascular plaques was alleviated in TPST1^−/−^/ApoE^−/−^ and TPST2^−/−^/ApoE^−/−^ mice (Fig. 4C + D). These results further confirmed the correlations between sulfated monocytes/macrophages and CKD-related atherosclerosis, and that sulfated monocytes/macrophages participate in the formation of CKD-related atherosclerosis.

### Sulfation inhibition decreases peritoneal macrophage adherence and migration

**Fig. 5:**
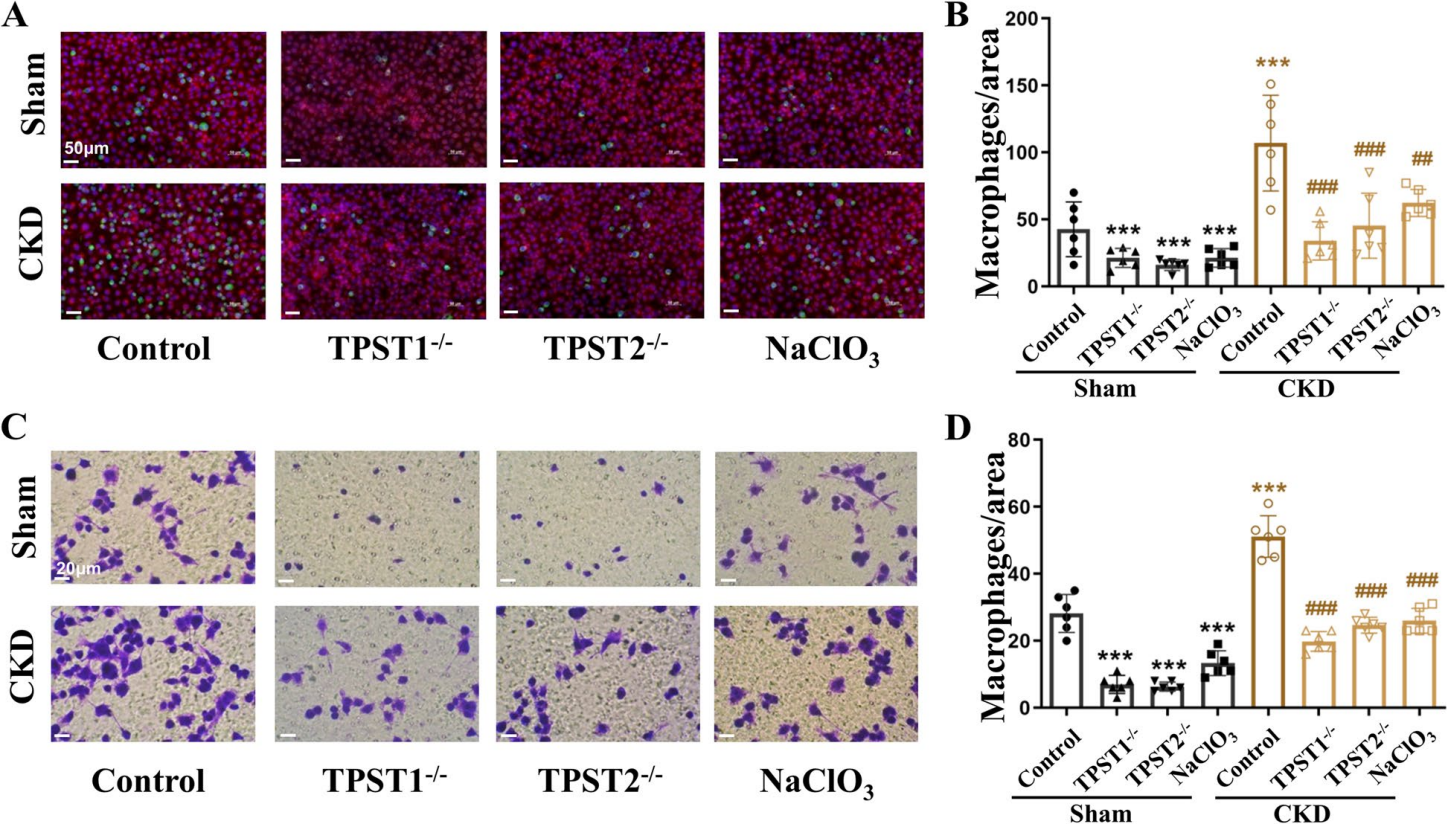
Sulfation inhibition attenuates adherence and migration of peritoneal macrophages. **A** CKD by 5/6 nephrectomy stimulated adherence of peritoneal macrophages to endothelial cells compared with the sham group (Control). This effect was significantly decreased after knockout of TPST1 (TPST1^−/−^) and TPST2 (TPST2^−/−^). Similar phenomenon was observed in the groups treated with the sulfation inhibitor sodium chlorate (NaClO_3_). Scale bar: 50 μm. **B** Quantitative analysis for cells adherence in different groups. Adherent macrophages were counted with a fluorescence microscope. *n* = 6 in each group. **C** CKD by 5/6 nephrectomy promoted peritoneal macrophage migration activity. Macrophage migration was decreased in cells from TPST1^−/−^ and TPST2^−/−^ mice or those treated with sodium chlorate (NaClO_3_). Scale bar: 20 μm. **D** Quantitative analysis for cell migration in different groups. The migration was quantified by blind counting of the migrated cells on the lower surface of the Boyden chamber membrane. *n* = 6 in each group. ^***^p < 0.001 compared with the control group, ^##^*p* < 0.01 compared with the CKD group, ^###^*p* < 0.001 compared with the CKD group. TPST, tyrosylprotein sulfotransferases

Monocytes adherence to endothelial cells is considered a significant pathogenetic step in atherosclerosis [24]. Thus, we further explored the migration and adhesion activities of peritoneal macrophages under TPST deficiency. Peritoneal macrophages from mice subjected to 5/6 nephrectomy showed enhanced adherence to endothelium. However, peritoneal macrophages from TPST1^−/−^ and TPST2^−/−^ mice adhered less (Fig. 5A + B). Employing the classic Boyden chambers assay, we found that peritoneal macrophages from mice subjected to 5/6 nephrectomy had increased migratory activity, which was reduced by TPST1 or TPST2 deficiency (Fig. 5C + D). Moreover, the adhesion and migration of macrophages were prohibited by sodium chlorate (NaClO_3_), the inhibitor of sulfation.

### Sulfation of CCR2 and CCR5 is increased in PBMCs from ESRD patients

**Fig. 6:**
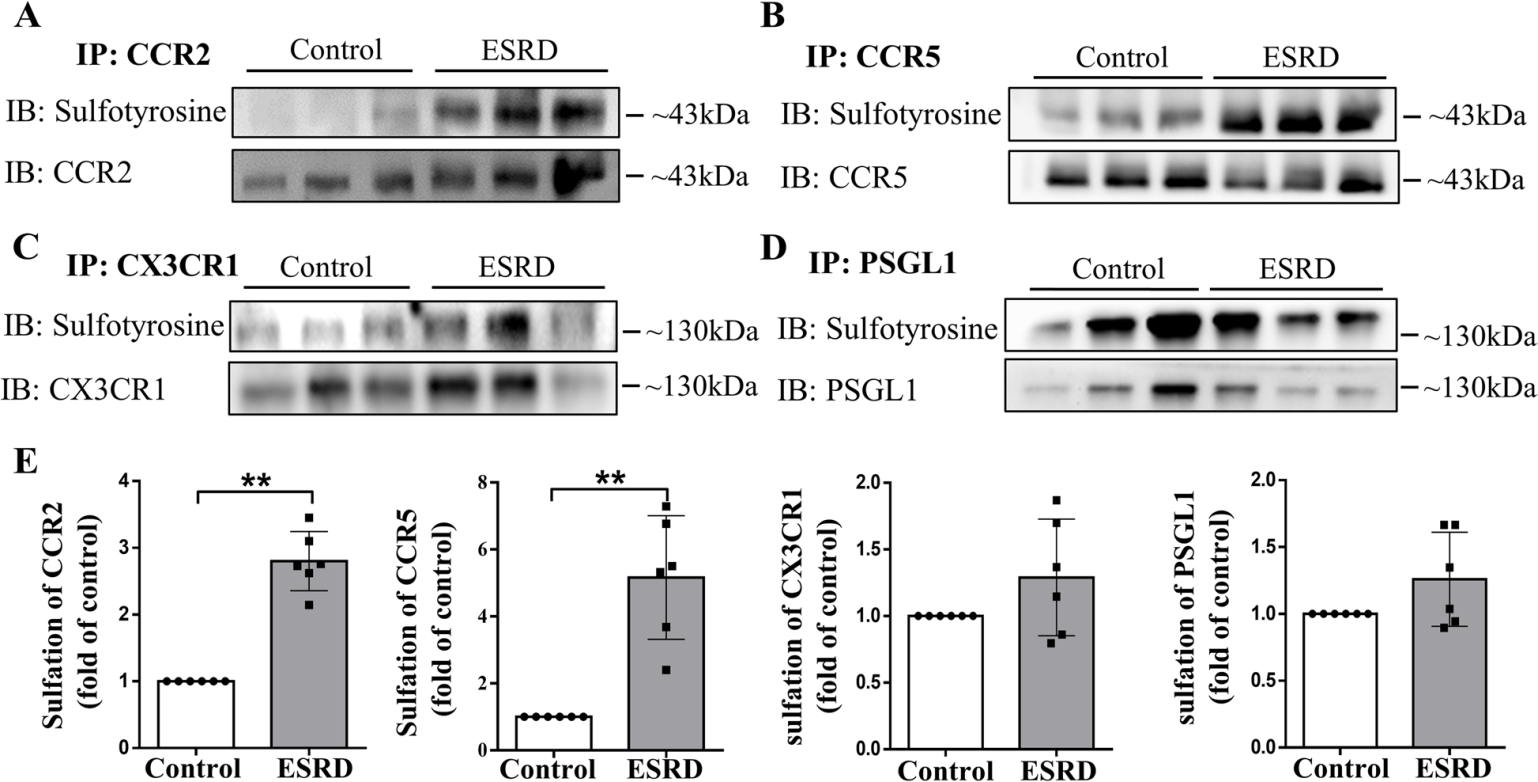
Increased sulfation of chemokine receptors in PBMCs from individuals with ESRD. **A**–**D** Western blot analyses of sulfation level of CCR2, CCR5, CX3CR1, and PSGL1 in PBMCs extracted from ESRD patients and control subjects. The results showed that these chemokine receptors were all over-sulfated, among which CCR2 and CCR5 reached statistical differences. Band densitometry was quantified in E. ^**^P < 0.01 compared with the control group

To further investigate the underlying mechanism of tyrosine sulfation on CKD-related atherosclerosis, PBMCs from the study cohort 1 ESRD patients were collected, and the sulfation status of the main adhesion molecules and chemokine receptors, including CCR2, CCR5, CX3CR1, and PSGL1, was determined. Immunoprecipitation and western blot analyses showed that all of the adhesion molecules and chemokine receptors were over-sulfated, among which CCR2 and CCR5 reached statistical differences (Fig. 6). These data indicate that more abundant infiltration of monocytes/macrophages in CKD-related atherosclerosis may be related to excessive sulfation of adhesion molecules and chemokine receptors.

## Discussion

**Fig. 7:**
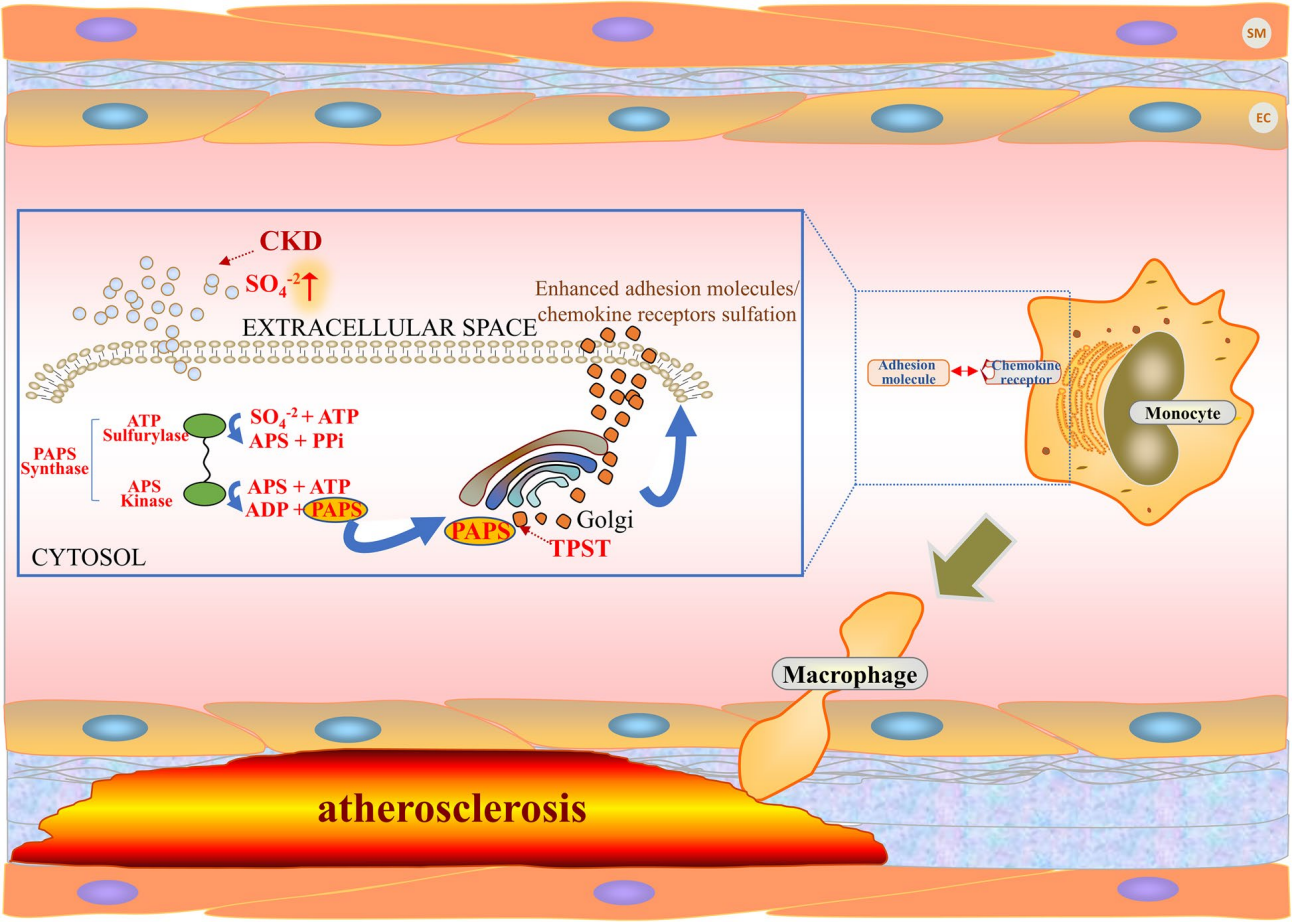
Correlations between enhanced tyrosine sulfation and CKD-related atherosclerosis. Chronic kidney disease (CKD) is associated with increased levels of inorganic sulfate (SO_4_^−2^) and the consequently enhanced tyrosine sulfation, which leads to the increased sulfation of specific adhesion molecules and chemokine receptors, including CCR2 and CCR5. This contributes to monocytes/macrophages activation, finally favoring the process of CKD-related atherosclerosis

To the best of our knowledge, this is the first report to describe increased sulfation in CKD and to link this to CKD-related atherosclerosis. The results showed that CKD could lead to increased inorganic sulfate level and the consequently enhanced tyrosine sulfation of specific adhesion molecules and chemokine receptors, including CCR2 and CCR5, which might be associated with monocyte/macrophage activation and promoted CKD-related atherosclerosis (Fig. 7). This mechanism was further confirmed by the clinical study, which revealed that O-sulfotyrosine, the metabolic end product of tyrosine sulfation, was significantly associated with the severity of coronary atherosclerosis assessed with SYNTAX score in CKD subjects. All of the enrolled CKD patients in our study underwent coronary angiography. These data are rare as CKD patients are usually hesitant to receive coronary angiography due to the potential adverse renal effects from contrast agents. In order to reduce the interference of additional atherosclerosis risk factors, patients with diabetes mellitus, familial hypercholesterolemia, and systemic disease were excluded, making the results more convincing.

The pathophysiology of atherosclerosis is very complex and involves many cell types, among which monocytes are the main subtype that migrates toward atherosclerotic lesions and differentiates into macrophages [25, 26]. Specifically, the process of monocytes extravasation involves a weak attachment and rolling of monocytes along the endothelium, followed by the activation of monocytes, firm adhesion and transmigration of monocytes through the endothelial layer to the underlying tissue by a process consisting of various interactions between adhesion molecules and chemokines [27]. Chemokine receptors (such as CCR2, CCR5, and CX3CR1) and adhesion molecules (such as PSGL-1) have been reported to be needed for monocytes recruitment toward atherosclerotic lesions [28–31]. Tyrosine sulfation, which occurs in the trans-Golgi network, is a common PTM of the membrane and secreted proteins that enhances the interaction with other molecules [32]. Chemokine receptors and adhesion molecules, including CCR2, CCR5, CX3CR1, and PSGL-1, have been repeatedly demonstrated to take an important part among the proteins undergoing tyrosine sulfation, which leads to the enhancement of their interaction functions and constitutive involvement in the trafficking and recruitment of monocytes/macrophages [13, 33]. In the present study, the effect of sulfation on monocyte/macrophage function under CKD condition was investigated. We found the increased infiltration of sulfated macrophages in the aortic plaques after CKD establishment, as well as the increased percentage of sulfate-positive nucleated cells in peripheral blood. In ex vivo study, decreased peritoneal macrophage adherence and migration activities were revealed after targeting tyrosine sulfation under CKD condition. In depth, increased sulfation levels of CCR2 and CCR5 were confirmed by immunoprecipitation in PBMCs purified from CKD patients. These results indicate that monocyte/macrophage function is enhanced via excessive sulfation of particular adhesion molecules and chemokine receptors in CKD condition, which would finally promote atherosclerosis. As more proteins have been shown to undergo tyrosine sulfation, further investigation is needed to clarify whether there are more adhesion molecules and chemokine receptors that participate in sulfation process. Enrichment of proteins with anti-sulfation antibodies and identification of specific proteins and tyrosine residues with mass spectrometry (MS) method would provide evidence. It should also be noted that adhesion molecules and chemokine receptors are widely expressed and are prominently present on various cells that play a crucial role in atherosclerosis development, including endothelial cells, smooth muscle cells, and leukocytes [34]. The effects of sulfation on these cells need further investigation.

Since its first finding in 1954 [35], protein tyrosine sulfation has been considered a widespread PTM that occurs on up to 1% of all tyrosine residues [11]. Available findings on tyrosine sulfation include the revelation of structure of the main tyrosine sulfation enzymes TPST1 and 2 [36, 37], the phenotype of TPST1 and 2 gene deficiency [38–40], the determination of TPST inhibitors [41–43], and the development of software that predicts tyrosine sulfation sites in protein sequences [44, 45]. Recently, synthetic sulfopeptides and sulfoproteins have also been used to reveal the functional roles of tyrosine sulfation [46–48]. However, there are still unresolved challenges in identifying and characterizing the function of tyrosine sulfation, resulting in the incomplete understanding of the full scope of its role. For instance, although MS has been considered an essential tool for analyzing PTMs in proteins, and many research groups have reported optimized methods to detect tyrosine sulfation by MS [49–54], many tyrosine sulfation sites were still missed, as tyrosine sulfate ester is sensitive to acid, heat, and high-energy ionization techniques during protein isolation and analysis procedures [55]. Moreover, unlike many other PTMs that degrade into identifiable markers, the study of tyrosine sulfation by MS is further complicated by the degradation product being native tyrosine [56]. Instead, detecting sulfation with anti-sulfotyrosine antibody would provide a supplemental method.

Gene deficiency of the main tyrosine sulfation enzymes TPST1 and 2 provides another effective method to study the effect of tyrosine sulfation. In mice, double deficiency of TPST1 and TPST2 led to postnatal pulmonary failure and death [57]. Thus, in our study, TPST1 and TPST2 knockout mice were separately established. Though both TPST1 and TPST2 knockouts were shown to decrease tyrosine sulfation, the importance of each of them and their combination remains to be determined. Independent of kidney injury, we found that loss of TPST1 and TPST2 decreased aortic atherosclerosis in ApoE^−/−^ mice. After 5/6 nephrectomy, the inhibitory effect of TPST deficiency seemed to be enhanced. In the absence of ApoE, cholesterol-rich lipoproteins accumulate in arteries and are taken up by leukocytes, including monocytes/macrophages. Foam cells then develop and contribute to atherosclerosis [58]. Under CKD condition, excessive sulfation would enhance the activities of monocytes/macrophages and contribute to the process of atherosclerosis. In contrast, in TPST deficiency, the function of leukocytes including monocytes/macrophages would be strongly attenuated due to the insufficient sulfation of adhesion molecules and chemokine receptors in these cells, finally leading to the attenuation of atherosclerosis. It should be noted that the gene deficiency method has its own disadvantages, including the potential influence of decreased body weight on atherosclerotic plaque formation. Kevin Moore’s group first reported reduced body weight both in TPST1- and TPST2-deficient mice [38, 40]. Our study found similar phenomenon and showed that both TPST1- and TPST2-deficient mice had significantly decreased body weight compared with wild-type mice, while TPST2-deficient mice have a greater reduction in body weight (Additional File 7: Fig. S6). In our study, the aortic lesion size was quantified by computerized image analysis after staining the entire aorta with Oil red O. The percentage of lesion coverage was calculated by dividing the stained area by the total thoracoabdominal aortic surface area, which would decrease the potential influence of body weight on atherosclerosis formation.

In mammals, sulfate homeostasis is regulated by the kidneys. Most of the filtered sulfate is absorbed in the proximal tubules, with a minority excreted in the urine [59]. Plasma inorganic sulfate levels are markedly increased in CKD [60], and some might participate in the formation of uremic toxins such as p-cresol sulfate and indoxyl sulfate [61]. Though the increased sulfation status found in CKD condition could be partly explained by the decreased elimination after kidney dysfunction, inorganic sulfate might provide excess substrate for sulfation and thereby cause the high sulfation status in CKD [20]. The positive correlation between plasma inorganic sulfate and O-sulfotyrosine levels revealed in the present study support this hypothesis.

## Conclusions

Although accelerated atherosclerosis in CKD has been demonstrated repeatedly in clinical and experimental studies, the treatment for atherosclerosis patients with CKD is the same as that for patients with normal kidney function. Specific treatment strategies are urgently needed to improve the outcomes of atherosclerosis in CKD condition. Our study indicated that the increased sulfation in CKD patients could enhance monocyte/macrophage activation and promote atherosclerosis. This study revealed a new potential mechanism contributing to CKD-related atherosclerosis and may provide a new target for the treatment of atherosclerosis in CKD.

## Methods

### Study population

First, in order to investigate the sulfation status in CKD patients, blood samples were obtained from patients with end-stage renal disease (ESRD) and controls in the study cohort 1. A total of 81 patients with CKD on maintenance hemodialysis (glomerular filtration rate [GFR] < 15 mL/min/1.73 m^2^) who were treated from March 2017 to April 2017 at the Department of Nephrology, Ruijin Hospital, were involved. Ninety patients with GFR ≥ 60 mL/min/1.73 m^2^ served as a control group. GFR was estimated using the Chronic Kidney Disease Epidemiology Collaboration (CKD-EPI) formula [62]. Blood samples from ESRD patients and controls were all collected after an overnight fasting. For ESRD patients, blood samples were collected on the day after hemodialysis treatment. PBMCs from ESRD patients and controls were then purified by Ficoll density-gradient centrifugation (1.077 g/mL, Sigma, MO, USA). Proteins were extracted from recovered PBMCs using a ProteoJET Mammalian Cell Lysis Reagent (Thermo Fisher Scientific, MA, USA). Plasma samples were also obtained in both groups by centrifuging blood samples at 3,500 g for 15 min at 4 °C. Plasma biochemical parameters, including sulfate levels, were measured. The expression levels of total sulfotyrosine, TPST1, and TPST2 in PBMCs, and the expression level of total sulfotyrosine in plasma were analyzed by western blot. Next, the sulfate status of adhesion molecules and chemokine receptors from the same PBMCs samples, including PSGL1, CCR2, CCR5, and CX3CR1, was studied by co-immunoprecipitation (Co-IP).

Second, in order to determine the association between sulfation status and atherosclerosis severity, the study cohort 2 included 107 patients with CKD (defined as GFR < 60 mL/min/1.73 m^2^ for more than three months) admitted to the Department of Vascular & Cardiology, Ruijin Hospital, between January 2013 and December 2019. Each patient underwent a coronary angiograph using the Judkins technique in multiple angulated views, and the SYNTAX score was evaluated. Patients were excluded if there was a previous history of myocardial infarction, coronary artery bypass graft (CABG) surgery, and/or percutaneous coronary intervention (PCI). Patients were also excluded if they had diabetes mellitus, malignant tumor, familial hypercholesterolemia, systemic disease (e.g., systemic lupus erythematosus), and/or left ventricular (LV) systolic dysfunction defined as an LV ejection fraction ≤ 50%. The underlying renal diseases in the study cohort included hypertensive nephropathy (*n* = 42), chronic glomerulonephritis (*n* = 29), chronic interstitial nephritis (*n* = 4), polycystic kidney disease (*n* = 2), and other/unknown renal diseases (*n* = 30). Blood samples were collected after an overnight fast, and plasma biochemical parameters, including sulfate levels, were measured. The levels of O-sulfotyrosine, the metabolic end product of tyrosine sulfation, were determined by liquid chromatography-mass spectrometry (LC–MS, Agilent Technologies, CA, USA). The relationships between SYNTAX score and plasma biochemical parameters, including O-sulfotyrosine, were analyzed.

### Coronary angiographies and SYNTAX scores

Patients underwent coronary angiographies through the radial artery using the Judkins technique. Each coronary artery was displayed in at least two different planes. All coronary angiograms were recorded on compact disks in DICOM format and were assessed independently by two experienced interventional cardiologists who were blinded to the study protocol and patient characteristics. In the case of disagreement, the opinion of a senior interventional cardiologist was sought, and the final decision was made by consensus. The SYNTAX score for each patient was calculated based on all coronary lesions with a stenosis of ≥ 50% of the diameter in vessels with a segment length > 1.5 mm using the SYNTAX score algorithm available on the SYNTAX website (www.syntaxscore.com) [63].

### Biochemical investigation

Blood samples from all participants were collected after overnight fasting. Biochemical data, including white blood cells (WBC), red blood cells (RBC), platelets (PLT), hemoglobin (HGB), alanine aminotransferase (ALT), aspartate aminotransferase (AST), albumin (ALB), creatinine (CRE), blood urea nitrogen (BUN), uric acid (UA), cystatin C (CysC), total cholesterol (TC), triglycerides (TG), low-density lipoprotein cholesterol (LDL-C), high-density lipoprotein cholesterol (HDL-C), and high-sensitivity C-reactive protein (hsCRP), were measured using standard laboratory techniques.

Plasma sulfate level was determined with a commercially available kit (MAK132, Sigma, MO, USA) according to the manufacturer’s protocol. Briefly, the plasma sample was deproteinated by protein precipitates on a table centrifuge, and supernatant was transferred for assay. After mixing with a master reaction mix, the sulfate level was measured by the spectrophotometric method (Hach, CO, USA) at 600 nm.

### Plasma O-sulfotyrosine determination

Plasma levels of O-sulfotyrosine were analyzed using a high-performance liquid chromatography (HPLC) system and mass spectrometer (Agilent Technologies, CA, USA) in line with the method reported previously [64]. Briefly, plasma was collected, and 1.5 mL of chloroform/methanol (2:1, v/v) was added. The solution was vortexed for 1 min and then centrifuged for 10 min at 3,000 g. Next, 800 μL of the organic phase was added to a clean tube, and the organic phase was dried with nitrogen. After adding 200 μL of isopropanol/methanol solution (1:1, v:v), the supernatant was transferred to HPLC vials for LC–MS analysis. A Waters UPLC BEHC18 column (2.1 mm × 50 mm, 1.7 μm) was used for chromatographic separation. Multiple reaction monitoring mode was used to detect O-sulfotyrosine.

### Animal studies

Wild-type and ApoE-null mice (ApoE^−/−^) were purchased from the Model Animal Research Center of Nanjing University. TPST1- and TPST2-knockout mice (TPST1^−/−^ & TPST2^−/−^) were established and then hybridized with ApoE^−/−^ mice to generate TPST1^−/−^/ApoE^−/−^ and TPST2^−/−^/ApoE^−/−^ mice (Model Organisms, Shanghai, China). Only male mice were included in the study, and all of the animals were maintained in a pathogen-free facility in the Animal Experiment Center of Ruijin Hospital, Shanghai Jiao Tong University School of Medicine. ApoE^−/−^ mice were given a high-cholesterol diet (21% fat, 0.2% cholesterol, 23% proteins, and 40.5% carbohydrates; Research Diets, NJ, USA) for 12 weeks to form atherosclerotic plaques.

### 5/6 nephrectomy

The CKD model was induced by a two-step 5/6 nephrectomy as described [65]. Briefly, 8-week-old experimental mice were anesthetized with 1.5% pentobarbital sodium, and the upper and lower poles of the right kidney were resected through a 2 cm flank incision. Left nephrectomy was carried out through a similar incision one week later. Special care was taken to avoid damage to the adrenal glands. Sham surgery was performed in the same way as described for CKD, except for the manipulation of the kidneys.

### TPST1- and TPST2-knockout study

CRISPR/Cas9-mediated TPST1^−/−^ and TPST2^−/−^ mice on a C57BL/6 background were produced by introducing a frame shift into the open reading frame (ORF) of the target genes through non-homologous end joining, which finally caused a loss of gene function (Model Organisms, Shanghai, China). Briefly, Cas9 mRNA and gRNA were obtained through in vitro transcription and then microinjected into the fertilized eggs of C57BL/6 J mice to generate F0 mice. The heterozygotes among F0 mice were identified by gene sequencing and mated with C57BL/6 J mice to obtain F1 generation mice.

The Ensemble No. for TPST1 was ENSMUSG00000034118 (see http://asia.ensembl.org/Mus_musculus/Gene/Summary?db=core;g=ENSMUSG00000034118;r=5:130073326-130135729;t=ENSMUST00000118993), and the exon 2–3 of transcript variant TPST1-202 (ENSMUST00000118993.7) was targeted for removal (Additional File 8: Fig. S7A). gRNA for TPST1 gene sequence was 5’-CACCATGGAAAGACTCACCA-3’ and 5’-AGTGGTCCTTACACTTAAGG-3’. Heterozygous F0 mice lost the 13,776 base pair as shown in Additional File 9: Table S2. The heterozygous F0 mice were bred with wild-type C57BL/6 J mice to obtain the F1 generation. The heterozygous state of F1 mice was confirmed by polymerase chain reaction (PCR) analysis using genomic DNA extracted from the mice tails (Additional File 8: Fig. S7B). Primers (P1–P4) are listed in Additional File 10: Table S3. The mutant mice were backcrossed with the wild-type line C57BL/6 mice for more than five generations to generate TPST1^−/−^ homozygotes for experimental use. The genotypes of the mutant mice were determined by PCR analysis using genomic DNA isolated from the mouse tail with the primers for wild-type allele (P3 and P4) and TPST1 mutant allele (P1 and P2). Product lengths of the wild-type and TPST1 mutant alleles were 724 bp and 601 bp, respectively (Additional File 8: Fig. S7C + D).

The Ensemble No. for TPST2 was ENSMUSG00000029344 (see http://asia.ensembl.org/Mus_musculus/Gene/Summary?db=core;g=ENSMUSG00000029344;r=5:112276691-112315361;t=ENSMUST00000031287), and the exon 3–6 of TPST2-201 (ENSMUST00000031287.10) was targeted for removal (Additional File 11: Fig. S8A). gRNA for the TPST2 gene sequence was 5’-AGGCCTGTAAGCAAGCTACT-3’ and 5’-GGCTGTACCATGTATCTGTG-3’. Heterozygous F0 mice lost the 6273 base pair as shown in Additional File 12: Table S4. Heterozygous F0 mice were bred with wild-type C57BL/6 J mice to obtain the F1 generation. The heterozygous state of F1 mice was confirmed by PCR analysis using genomic DNA extracted from the mice tails (Additional File 11: Fig. S8B). Primers (P5–P8) are listed in Additional File 10: Table S3. The mutant mice were backcrossed with the wild-type line C57BL/6 mice for more than five generations to generate TPST2^−/−^ homozygotes for experimental use. The genotypes of the mutant mice were determined by PCR analysis using genomic DNA isolated from the mouse tail with the primers for control (P9 and P10), wild-type allele (P7 and P8), and TPST2 mutant allele (P5 and 6). Product lengths of the control, wild-type, and TPST2 mutant alleles were 200 bp, 484 bp, and 805 bp, respectively (Additional File 11: Fig. S8C + D).

The heterozygous TPST1- and TPST2-mutant mice were backcrossed with ApoE^−/−^ mice to produce TPST1^−/−^/ApoE^−/−^ and TPST2^−/−^/ApoE^−/−^ mice. The genotypes of TPST1^−/−^/ApoE^−/−^ mutant mice were determined by PCR analysis using genomic DNA isolated from the mouse tail with the primers for control (P9 and P10), TPST1 wild-type allele (P3 and P4), TPST1 mutant allele (P1 and P2), APOE wild-type allele (P11 and P12), and APOE mutant allele (P11 and P13). Product lengths of the control, TPST1 wild-type allele, TPST1 mutant allele, APOE wild-type allele, and APOE mutant allele were 200 bp, 724 bp, 601 bp, 155 bp, and 245 bp, respectively (Additional File 13: Fig. S9A). The genotypes of TPST2^−/−^/ApoE^−/−^ mutant mice were determined by PCR analysis with the primers for control (P9 and P10), TPST2 wild-type allele (P7 and P8), TPST2 mutant allele (P5 and P6), APOE wild-type allele (P11 and P12), and APOE mutant allele (P11 and P13). Product lengths of the control, TPST2 wild-type allele, TPST2 mutant allele, APOE wild-type allele, and APOE mutant allele were 200 bp, 484 bp, 805 bp, 155 bp, and 245 bp, respectively (Additional File 13: Fig. S9B).

Mice were then subjected to 5/6 nephrectomy. After 12 weeks of high-cholesterol diet, the aorta was dissected, and an aortic lesion en-face assay was performed by staining with Oil red O. The percentage of lesion coverage was calculated by dividing the stained area by the total aortic surface area.

### Peritoneal macrophage adhesion and migration study

Experimental mice were divided into eight groups. Group 1 represented wild-type C57/BL mice with sham operation (Control), Group 2 represented wild-type C57/BL mice with 5/6 nephrectomy (CKD), Group 3 represented TPST1^−/−^ mice with sham operation (TPST1^−/−^), Group 4 represented TPST1^−/−^ mice with 5/6 nephrectomy (TPST1^−/−^ + CKD), Group 5 represented TPST2^−/−^ mice with sham operation (TPST2^−/−^), Group 6 represented TPST2^−/−^ mice with 5/6 nephrectomy (TPST2^−/−^ + CKD), Group 7 represented sham-operated mice with TPST inhibitor NaClO_3_ treatment (NaClO_3_), and Group 8 represented 5/6 nephrectomy mice with NaClO_3_ treatment (NaClO_3_ + CKD). Mice primary peritoneal macrophages were isolated with the method reported previously [66], and macrophage-endothelium interactions and migration activity were investigated subsequently. For NaClO_3_ treatment, peritoneal macrophages were isolated and treated with 30 mM NaClO_3_ for 24 h [67].

Mice primary peritoneal macrophages-endothelial cell adhesion assays were performed as published [68]. Briefly, the mice were anesthetized by intraperitoneal injection of 1.5% pentobarbital sodium, and 5 mL sterile thioglycolate medium (4%, BD, Detroit, MI, USA) was injected intraperitoneally. Diluted peritoneal fluid was harvested and loaded onto a 70-μm cell strainer (Corning, NY, USA). The filtrate was centrifuged at 250 g for 15 min. The cell pellet was suspended and incubated at 37 °C in a humidified incubator at 5% CO_2_ in Dulbecco's modified Eagle's medium (DMEM) supplemented with 10% (vol/vol) fetal bovine serum (FBS) (Gibco, MD, USA), 100 units/mL penicillin, and 100 μg/mL streptomycin (Gibco, MD, USA). Human umbilical vein endothelial cells (HUVECs) were seeded in 6-well tissue culture plates and grown for 48–72 h. Peritoneal macrophages were harvested and incubated in each of the HUVECs plates buffered with an equal volume of endothelial basal medium/1% FBS at 37 °C. After 24 h of adsorption, the plate was washed twice with Hank's balanced salt solution (HBSS) to remove nonadherent macrophages and fixed with 10% buffered formalin for 30 min at 4 °C. Cell nuclei were stained with Hoechst 33,342, and the adherent macrophages were quantified by a fluorescence microscope at a magnification of × 200 with a fluorescein isothiocyanate-based filter. Macrophage-endothelium cell adhesion was quantified as the number of adhering macrophages per area.

Peritoneal macrophage migration capacity was assessed using 8-μm pore transwell chambers (Corning, NY, USA). In brief, peritoneal macrophages were harvested and seeded into the upper chamber in DMEM (5 × 10^5^/well), while the lower chamber was filled with conditioned medium containing 10% FBS. The cells on the top filter were removed after 24 h, and the cells on the bottom filters were fixed with 4% paraformaldehyde for 10 min at room temperature. The number of cells that migrated across the filter was counted by rhodamine phalloidin staining of at least 6 fields per chamber using an optical microscope (Olympus, San Jose, CA).

### Histology and immunostaining

In general, experimental mice were fasted overnight and anesthetized with 1.5% pentobarbital sodium. Mice were perfused with ice-cold normal saline, and the heart and arteries were dissected out. The aortic lesion size was quantified by computerized image analysis after staining the entire aorta with Oil red O (Sigma, MO, USA). The percentage of lesion coverage was calculated by dividing the stained area by the total thoracoabdominal aortic surface area.

In addition, serial sections of aortic sinus lesions were prepared by microtome cutting into 10 μm cryosections. The sections were stained with filtered Oil red O in a wet container, and lesion’s collagen was stained using a Masson-trichrome staining kit (Baso Inc., NY, USA). For plaque immunostaining, the sections were blocked with 10% normal serum for 30 min and then incubated with primary antibodies recognizing CD68 (Bio-Rad, Kidlington, UK), sulfotyrosine (Merck, Darmstadt, Germany), and F4/80 (AbDserotec, Oxford, UK) overnight at 4 °C. Next, the sections were washed and incubated with biotin-conjugated secondary antibody or fluorescent secondary antibodies for 60 min. The sections without primary antibody served as negative controls.

For bone marrow immunostaining, femurs were removed and fixed in 4% paraformaldehyde, decalcified in 10% EDTA for 5 to 7 days at 4 °C with the help of Shanghai Key Laboratory for Bone and Joint Disease, Shanghai Institute of Traumatology and Orthopaedics (Shanghai, China) [69]. Decalcified femurs were dehydrated and embedded in paraffin, after which 10-μm-thick sections were cut on a rotary microtome. The sections were used for immunostaining as described above.

To analyze the sulfation of the peripheral blood nucleated cells, 100 µL of anti-coagulated blood was obtained, and the cells were harvested with density centrifugation. The cells were then resuspended to the adhesion micro slide until sedimentation and fixed with 1% paraformaldehyde. The micro slides were air dried and stained with anti-sulfotyrosine antibody (Merck, Darmstadt, Germany). Images were captured with an Olympus microscope and quantitatively analyzed using Image-Pro Plus 6.0 (Roper Industries, CA, USA).

### Immunoprecipitation and western blot

Proteins from PBMCs and bone marrow samples were extracted with a ProteoJET Mammalian Cell Lysis Reagent (Thermo Fisher Scientific, MA, USA). Protein concentrations in PBMCs and bone marrow lysis, as well as plasma samples, were measured using the BCA Assay (Pierce, Thermo Fisher Scientific, MA, USA). Proteins samples were then used for immunoprecipitation and western blot analysis. Immunoprecipitation was carried out using Pierce Crosslink Immunoprecipitation Kit (Thermo Fisher Scientific, MA, USA) as instructed. Briefly, 10 µg of antibodies against CCR2 (CST, MA, USA), CCR5 (Abcam, MA, USA), CX3CR1 (Abcam, MA, USA), and PSGL1 (Abcam, MA, USA), and 500 µg of total proteins were used in immunoprecipitation reaction, followed by 1 h incubation with protein A/G plus agarose beads. The beads were then collected and washed six times with the lysis buffer, and proteins were eluted from the beads. For western blot analysis, equal amounts of proteins were subjected to SDS-PAGE and transferred to polyvinylidene fluoride membranes. The membranes were then blocked with 5% nonfat milk in TBST (tris-buffered saline with 0.2% Tween-20) for 1 h. The membranes were incubated overnight at 4 °C with primary antibodies against CCR2 (1:1000), CCR5 (1:1000), CX3CR1 (1:1000), PSGL1 (1:1000), sulfotyrosine (1:1000, Merck, Darmstadt, Germany), TPST1 (1:1000, Abcam, MA, USA), TPST2 (1:1000, Abcam, MA, USA), GM130 (1:1000, Abcam, MA, USA), and phosphotyrosine (1:1000, Abcam, MA, USA), washed three times, and incubated with secondary antibodies for 1 h at room temperature. The membranes were rinsed, and the signal was detected using an ECL detection system (Millipore, MA, USA).

### Statistical analysis

Data were analyzed using the SPSS 13.0 (SPSS Inc., Chicago, IL, USA). Continuous variables are presented as mean ± standard deviation (SD) and evaluated using Student’s *t* test between two groups. One-way analysis of variance followed by Bonferroni post-hoc analysis was performed to determine the differences among multiple groups. For categorical variables, we evaluated differences between the groups using the chi-square test. Pearson’s tests were used to evaluate the correlations between SYNTAX score and other variables. Multivariate logistic regression analysis was used for multivariate analysis of independent variables. A two-tailed *p* < 0.05 was considered statistically significant.

## Supplementary Information

**Additional file 1:** **Table S1.** Baseline clinical characteristics of control and ESRD patients in the study cohort 1. Data are shown as numberor mean ± SD. ESRD, end-stage renal disease; BMI, body mass index; WBC, white blood cells; RBC, red blood cells; HGB, hemoglobin; PLT, platelets; ALT, alanine aminotransferase; AST, aspartate aminotransferase; ALB, albumin; BUN, blood urea nitrogen; CRE, creatinine; UA, uric acid; CysC, cystatin C; GFR, glomerular filtration rate; TC, total cholesterol; TG, triglycerides; HDL-C, high-density lipoprotein cholesterol; LDL-C, low-density lipoprotein cholesterol; hsCRP, high-sensitivity C-reactive protein; LVEF, left ventricular rejection fraction.

**Additional file 2:** **Fig S1.** Bone marrow total sulfotyrosine, TPST1, and TPST2 expression levels in mice at different time points after 5/6 nephrectomy. Western blotting of bone marrow lysate solution revealed that the protein levels of total sulfotyrosine, TPST1, and TPST2 increased time-dependently after 5/6 nephrectomy. Three bone marrow tissues mixed in one lane, and quantitative measurement of western blot images as relative protein compared to β-actin were visualized. The experiments were repeated three times. Data are expressed as means ± SD. ^*^p<0.05 compared with the sham group. TPST, tyrosylprotein sulfotransferases.

**Additional file 3:** **Fig S2.** Bone marrow total sulfotyrosine, TPST1, and TPST2 expression levels in mice after 16 weeks of 5/6 nephrectomy. Total sulfotyrosine, TPST1, and TPST2 expression levels in bone marrow tissues after 16 weeks of 5/6 nephrectomy were detected by immunohistochemical staining, and showed a marked increase in total sulfotyrosine, TPST1, and TPST2 immunopositivity. Quantitative measurement of immunohistochemical images as the ratio of positive area showed that total sulfotyrosine, TPST 1, and TPST2 expression levels were highly induced after 5/6 nephrectomy. Scale bar: 100 μm, n=6 in each group. Data are expressed as means ± SD. ^***^p<0.001 compared with the sham group. CKD, chronic kidney disease; TPST, tyrosylprotein sulfotransferases.

**Additional file 4:** **Fig S3.** Correlations between plasma sulfate and O-sulfotyrosine levels. Pearson’s correlation coefficients showed that plasma sulfate levels positively correlated with O-sulfotyrosine concentrations in individuals with CKD. R^2^=0.4790, r=0.6921, *p*<0.001, Y=0.002×X+1.294. CKD, chronic kidney disease.

**Additional file 5:** **Fig S4.** Plasma sulfate levels in ApoE^-/-^ mice at different time points after 5/6 nephrectomy. ^***^p<0.001 compared with the shame group.

Additional file 6: **Fig S5.** The expression of total sulfotyrosine, TPST1, and TPST2 in TPST1^-/-^ and TPST2^-/-^ mice by western blot. The result showed that both TPST1 and TPST2 knockouts were able to significantly suppress tyrosine sulfation activities. TPST, tyrosylprotein sulfotransferases.

Additional file 7: **Fig S6**. Mean body weight of TPST1 gene deficiency, TPST2 gene deficiency, and wild-typemice. Both TPST1^-/-^ and TPST2^-/-^ mice showed decreased body weight compared with WT mice, but TPST2^-/-^ mice had a greater reduction in body weight. Data are expressed as means ± SD. ***p<0.001.

**Additional file 8:** **Fig S7.** Production and identification of TPST1^-/-^ mice.CRISPR/Cas9 strategy for TPST1 editing. The PCR primers for genotyping are indicated by arrows.PCR for F1 mice identification. HE: 601 bp +14377 bpamplified by P1 and P2, 724 bp amplified by P3 and P4.The genotypes of mutant mice were determined by PCR analysis using genomic DNA isolated from the mouse tail with the primers for WT alleleand TPST1 mutant allele. WT: A114, A120, A121; HE: A112, A115, A116, A118, A119, A122, A123; HO: A113, A117. M, 100 bp Plus DNA ladder. WT, wild type; HE, heterozygote; HO, homozygote; H_2_0, negative control.

**Additional file 9:** **Table S2.** Genotype identification results for F0 mice.

**Additional file 10:** **Table S3.** Primers for verification of vector construction.

**Additional file 11:** **Fig. S8.** Production and identification of TPST2^-/-^ mice.CRISPR/Cas9 strategy for TPST2 editing. The PCR primers for genotyping are indicated by arrows.PCR for F1 mice identification. HE: 805 bp + 7078 bpamplified by P1 and P2, 484 bp amplified by P3 and P4.The genotypes of mutant mice were determined by PCR analysis using genomic DNA isolated from the mouse tail with the primers for WT alleleand TPST2 mutant allele. WT: B50, B60, B61, B63, B64, B65, B67; HE: B52, B53, B54, B58, B59, B62; HO: B49, B51, B55, B56, B57, B66. M, 1 kb DNA ladder. WT, wild type; HE, heterozygote; HO, homozygote; H_2_0, negative control.

**Additional file 12:** **Table S4.** Genotype identification results for F0 mice.

**Additional file 13:** **Fig S9.** Identification of TPST1^-/-^/ApoE^-/-^ and TPST2^-/-^/ApoE^-/-^ mice.The genotypes of TPST1^-/-^/ApoE^-/-^ mutant mice were determined by PCR analysis using genomic DNA isolated from the mouse tail with the primers for control, TPST1 wild-type allele, TPST1 mutant allele, APOE wild-type allele, and APOE mutant allele. Product lengths of the control, TPST1 wild-type allele, TPST1 mutant allele, APOE wild-type allele, and APOE mutant allele were 200 bp, 724 bp, 601 bp, 155 bp, and 245 bp, respectively. A25 and A26 represented TPST1^+/-^/ApoE^-/-^.The genotypes of TPST2^-/-^/ApoE^-/-^ mutant mice were determined by PCR analysis with the primers for control, TPST2 wild-type allele, TPST2 mutant allele, APOE wild-type allele, and APOE mutant allele. Product lengths of the control, TPST2 wild-type allele, TPST2 mutant allele, APOE wild-type allele, and APOE mutant allele were 200 bp, 484 bp, 805 bp, 155 bp, and 245 bp, respectively. A231, A232, and A237 represented TPST2^+/-^/ApoE^-/-^, while A233, A234, A235, and A236 represented TPST2^+/+^/ApoE^-/-^. M, 100 bp plus DNA ladder. WT, wild type; MUT, mutation; H_2_0, negative control. TPST, tyrosylprotein sulfotransferases

## Data Availability

The datasets used and/or analyzed during the current study are included in this published article and its supplementary information files.

## Abbreviations

ALB: Albumin
ALT: Alanine aminotransferase
AST: Aspartate aminotransferase
BUN: Blood urea nitrogen
CABG: Coronary artery bypass graft
CKD: Chronic kidney disease
CKD-EPI: Chronic kidney disease epidemiology collaboration
Co-IP: Co-immunoprecipitation
CRE: Creatinine
CVD: Cardiovascular disease
CysC: Cystatin C
DMEM: Dulbecco’s modified Eagle’s medium
ESRD: End stage renal disease
FBS: Fetal bovine serum
GFR: Glomerular filtration rate
HBSS: Hank’s balanced salt solution
HDL-C: High-density lipoprotein cholesterol
HGB: Hemoglobin
HPLC: High-performance liquid chromatography
hsCRP: High-sensitivity C-reactive protein
HUVECs: Human umbilical vein endothelial cells
LC–MS: Liquid chromatography-mass spectrometry
LDL-C: Low-density lipoprotein cholesterol
LV: Left ventricular
MS: Mass spectrometry
NaClO_3_: Sodium chlorate
ORF: Open reading frame
PAPS: 3’-Phosphate 5’-phosphosulfate
PBMCs: Peripheral blood mononuclear cells
PCI: Percutaneous coronary intervention
PCR: Polymerase chain reaction
PCS: P-cresyl sulfate
PLT: Platelets
PTM: Post-translational modification
RBC: Red blood cells
SD: Standard deviation
TBST: Tris-buffered saline with 0.2% Tween-20
TC: Total cholesterol
TG: Triglycerides
TPST: Tyrosylprotein sulfotransferases
UA: Uric acid
WBC: White blood cells
