# Supplementary material for: Enhanced tyrosine sulfation is associated with chronic kidney disease-related atherosclerosis
Source: BMC Biol. 2023 Jul 10;21:151. doi: 10.1186/s12915-023-01641-y (PMC10332009; doi:10.1186/s12915-023-01641-y)
Supplement: Supplementary file 1 — Additional file 1: Table S1. Baseline clinical characteristics of control and ESRD patients in the study cohort 1. Data are shown as numberor mean ± SD. ESRD, end-stage renal disease; BMI, body mass index; WBC, white blood cells; RBC, red blood cells; HGB, hemoglobin; PLT, platelets; ALT, alanine aminotransferase; AST, aspartate aminotransferase; ALB, albumin; BUN, blood urea nitrogen; CRE, creatinine; UA, uric acid; CysC, cystatin C; GFR, glomerular filtration rate; TC, total cholesterol; TG, triglycerides; HDL-C, high-density lipoprotein cholesterol; LDL-C, low-density lipoprotein cholesterol; hsCRP, high-sensitivity C-reactive protein; LVEF, left ventricular rejection fraction. [file 12915_2023_1641_MOESM1_ESM.doc]

|  | Control Group  (n=90) | ESRD Group  (n=81) | *p* value |
| --- | --- | --- | --- |
| Male, n (%) | 59 (65.6%) | 51 (63.0%) | 0.724 |
| Age (years) | 64.4 ± 8.7 | 58.2 ± 13.3 | 0.001 |
| BMI (kg/m^2^) | 24.98±2.76 | 22.36±3.27 | 0.000 |
| Alcohol users (%) | 16 (17.8) | 0 (0) | 0.000 |
| Cigarette smoking (%) | 26 (28.9) | 2 (2.5) | 0.000 |
| Hypertension, n (%) | 61 (67.8%) | 64 (79.0%) | 0.098 |
| WBC (10^9^/L) | 6.23 ± 1.84 | 6.24 ± 2.02 | 0.994 |
| RBC (10^12^/L) | 4.42 ± 0.474 | 3.51 ± 0.620 | 0.000 |
| HGB (g/L) | 137.88 ± 14.994 | 106.09 ± 20.286 | 0.000 |
| PLT (10^9^/L) | 170.98 ± 60.106 | 151.27 ± 57.898 | 0.031 |
| ALT (IU/L) | 20.73 ± 7.07 | 20.83 ± 9.48 | 0.976 |
| AST (IU/L) | 31.59 ± 13.90 | 24.94 ± 10.03 | 0.270 |
| ALB (g/L) | 37.8 ± 2.2 | 33.3 ± 2.8 | 0.000 |
| BUN (mmol/L) | 8.42 ± 13.42 | 26.00 ± 5.65 | 0.000 |
| CRE (µmol/L) | 86.29 ± 54.21 | 499.14 ± 363.23 | 0.000 |
| UA (μmol/L) | 347.0 ± 80.3 | 379.4 ± 102.7 | 0.379 |
| CysC (mg/L) | 1.09 ± 0.18 | 7.06 ± 1.63 | 0.000 |
| GFR (mL/min/1.73m^2^) | 88.35 ± 16.76 | 4.27 ± 1.34 | 0.000 |
| Calcium (mmol/L) | 2.28 ± 0.08 | 2.25 ± 0.18 | 0.630 |
| Phosphate (mmol/L) | 1.17 ± 0.14 | 1.68 ± 0.43 | 0.000 |
| TC (mmol/L) | 4.20 ± 1.151 | 4.25 ± 1.091 | 0.786 |
| TG (mmol/L) | 1.64 ± 0.959 | 1.74 ± 1.101 | 0.528 |
| HDL-C (mmol/L) | 1.19 ± 0.264 | 1.09 ± 0.305 | 0.022 |
| LDL-C (mmol/L) | 2.44 ± 0.897 | 2.55 ± 0.859 | 0.426 |
| hsCRP (mg/l) | 2.95 ± 3.22 | 16.39 ± 18.51 | 0.016 |
| LVEF (%) | 64.86 ± 6.12 | 57.17 ± 8.67 | 0.028 |
| Plasma sulfate (mM) | 0.43 ± 0.211 | 2.37 ± 0.818 | 0.000 |
