# Supplementary figures and images for: Enhanced tyrosine sulfation is associated with chronic kidney disease-related atherosclerosis
Source: BMC Biol. 2023 Jul 10;21:151. doi: 10.1186/s12915-023-01641-y (PMC10332009; doi:10.1186/s12915-023-01641-y)

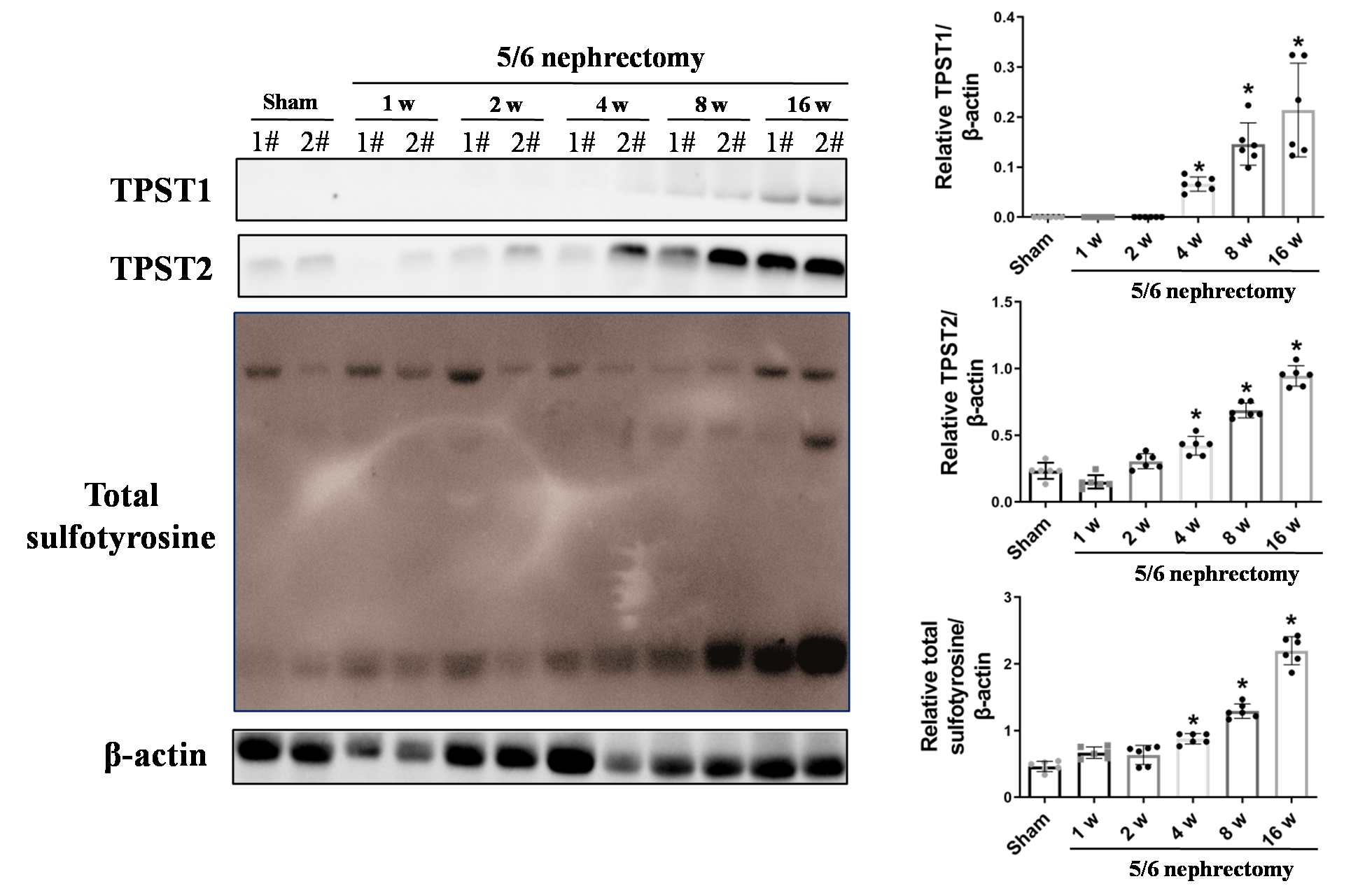

Supplement: Supplementary file 2 — Additional file 2: Fig S1. Bone marrow total sulfotyrosine, TPST1, and TPST2 expression levels in mice at different time points after 5/6 nephrectomy. Western blotting of bone marrow lysate solution revealed that the protein levels of total sulfotyrosine, TPST1, and TPST2 increased time-dependently after 5/6 nephrectomy. Three bone marrow tissues mixed in one lane, and quantitative measurement of western blot images as relative protein compared to β-actin were visualized. The experiments were repeated three times. Data are expressed as means ± SD. *p<0.05 compared with the sham group. TPST, tyrosylprotein sulfotransferases. [file 12915_2023_1641_MOESM2_ESM.tif]

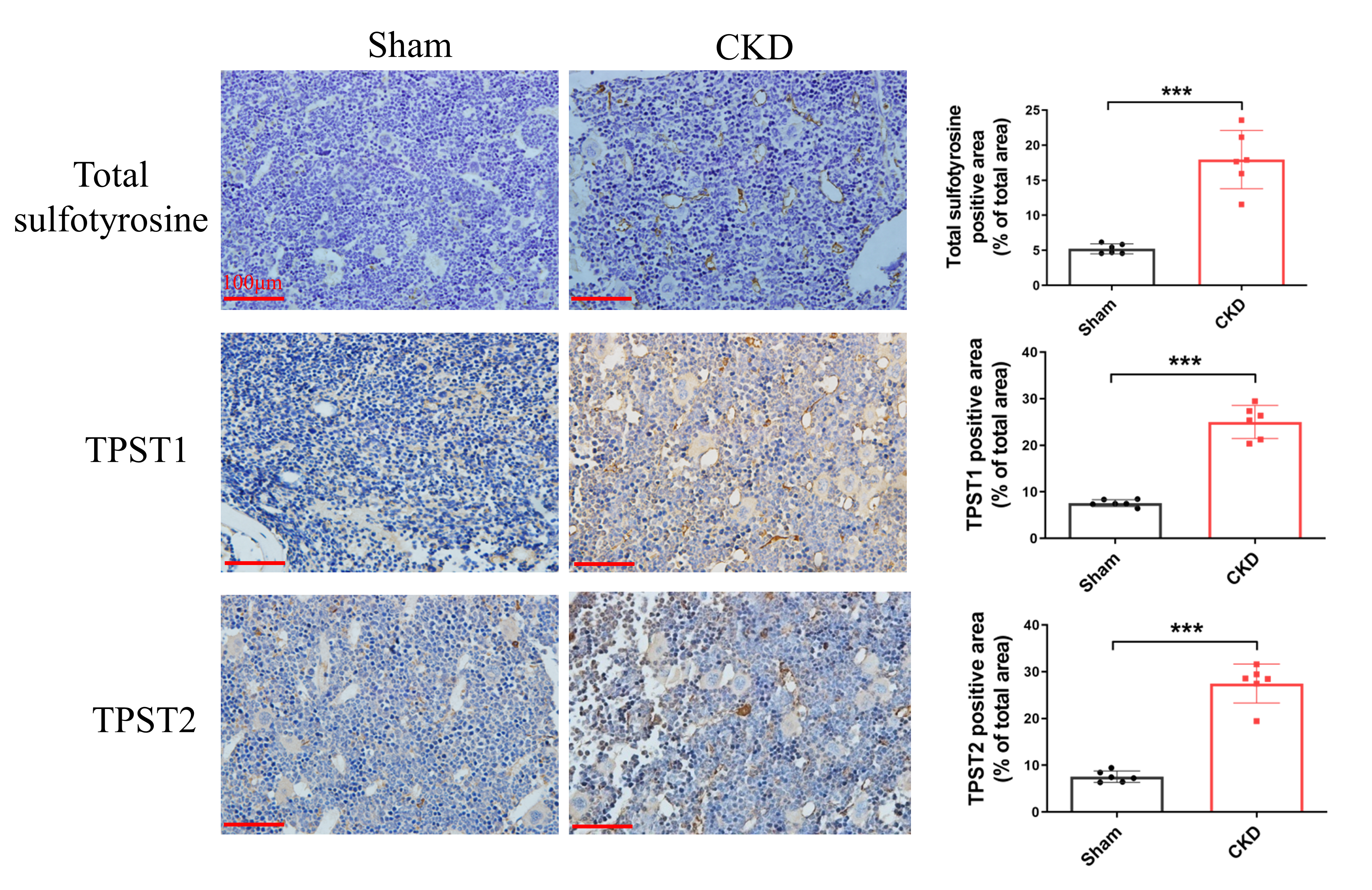

Supplement: Supplementary file 3 — Additional file 3: Fig S2. Bone marrow total sulfotyrosine, TPST1, and TPST2 expression levels in mice after 16 weeks of 5/6 nephrectomy. Total sulfotyrosine, TPST1, and TPST2 expression levels in bone marrow tissues after 16 weeks of 5/6 nephrectomy were detected by immunohistochemical staining, and showed a marked increase in total sulfotyrosine, TPST1, and TPST2 immunopositivity. Quantitative measurement of immunohistochemical images as the ratio of positive area showed that total sulfotyrosine, TPST 1, and TPST2 expression levels were highly induced after 5/6 nephrectomy. Scale bar: 100 μm, n=6 in each group. Data are expressed as means ± SD. ***p<0.001 compared with the sham group. CKD, chronic kidney disease; TPST, tyrosylprotein sulfotransferases. [file 12915_2023_1641_MOESM3_ESM.tif]

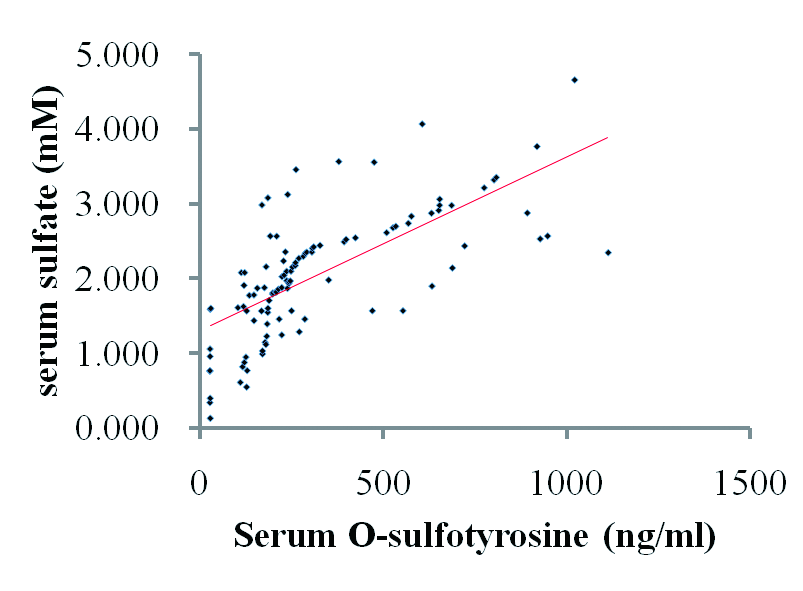

Supplement: Supplementary file 4 — Additional file 4: Fig S3. Correlations between plasma sulfate and O-sulfotyrosine levels. Pearson’s correlation coefficients showed that plasma sulfate levels positively correlated with O-sulfotyrosine concentrations in individuals with CKD. R2=0.4790, r=0.6921, p<0.001, Y=0.002×X+1.294. CKD, chronic kidney disease. [file 12915_2023_1641_MOESM4_ESM.tif]

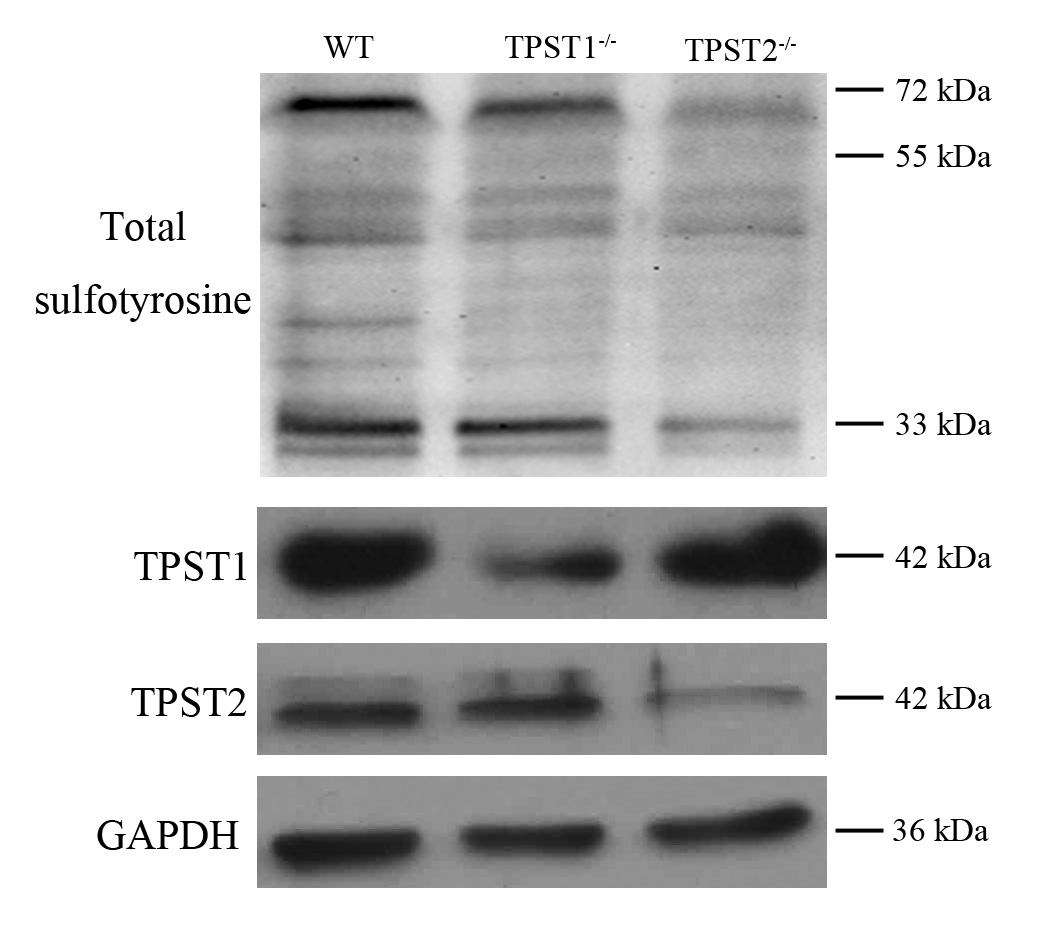

Supplement: Supplementary file 6 — Additional file 6: Fig S5. The expression of total sulfotyrosine, TPST1, and TPST2 in TPST1-/- and TPST2-/- mice by western blot. The result showed that both TPST1 and TPST2 knockouts were able to significantly suppress tyrosine sulfation activities. TPST, tyrosylprotein sulfotransferases. [file 12915_2023_1641_MOESM6_ESM.tif]

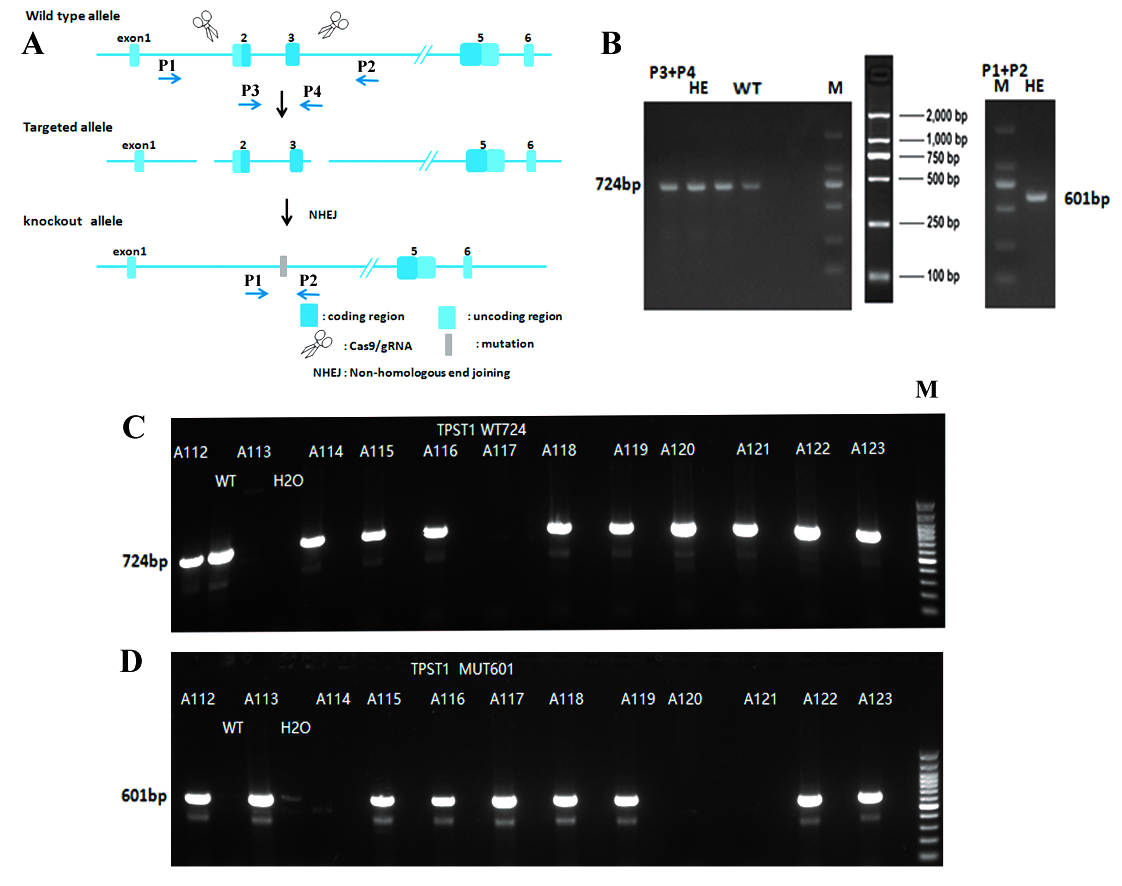

Supplement: Supplementary file 8 — Additional file 8: Fig S7. Production and identification of TPST1-/- mice.CRISPR/Cas9 strategy for TPST1 editing. The PCR primers for genotyping are indicated by arrows.PCR for F1 mice identification. HE: 601 bp +14377 bpamplified by P1 and P2, 724 bp amplified by P3 and P4.The genotypes of mutant mice were determined by PCR analysis using genomic DNA isolated from the mouse tail with the primers for WT alleleand TPST1 mutant allele. WT: A114, A120, A121; HE: A112, A115, A116, A118, A119, A122, A123; HO: A113, A117. M, 100 bp Plus DNA ladder. WT, wild type; HE, heterozygote; HO, homozygote; H20, negative control. [file 12915_2023_1641_MOESM8_ESM.tif]

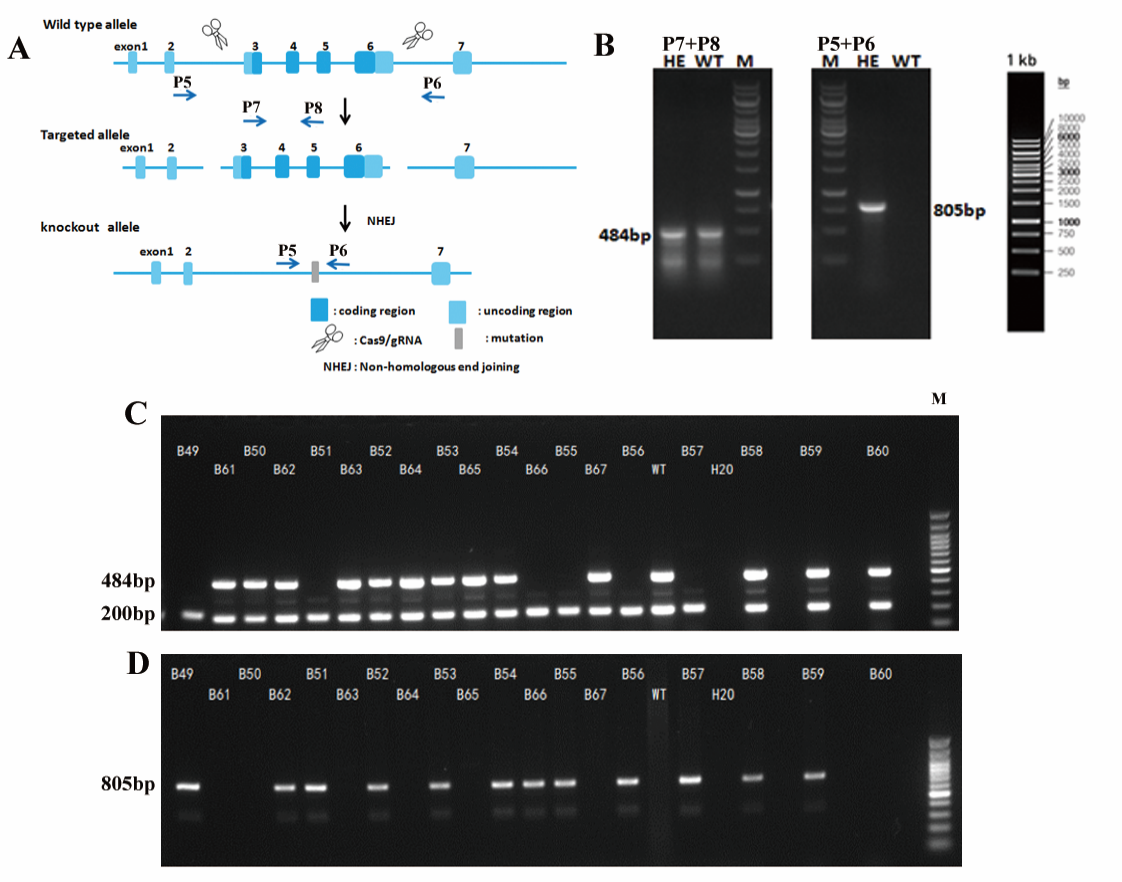

Supplement: Supplementary file 11 — Additional file 11: Fig. S8. Production and identification of TPST2-/- mice.CRISPR/Cas9 strategy for TPST2 editing. The PCR primers for genotyping are indicated by arrows.PCR for F1 mice identification. HE: 805 bp + 7078 bpamplified by P1 and P2, 484 bp amplified by P3 and P4.The genotypes of mutant mice were determined by PCR analysis using genomic DNA isolated from the mouse tail with the primers for WT alleleand TPST2 mutant allele. WT: B50, B60, B61, B63, B64, B65, B67; HE: B52, B53, B54, B58, B59, B62; HO: B49, B51, B55, B56, B57, B66. M, 1 kb DNA ladder. WT, wild type; HE, heterozygote; HO, homozygote; H20, negative control. [file 12915_2023_1641_MOESM11_ESM.tif]

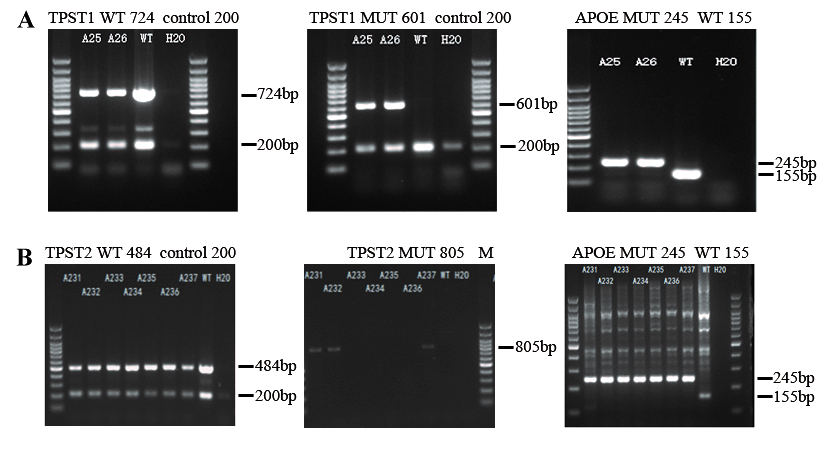

Supplement: Supplementary file 13 — Additional file 13: Fig S9. Identification of TPST1-/-/ApoE-/- and TPST2-/-/ApoE-/- mice.The genotypes of TPST1-/-/ApoE-/- mutant mice were determined by PCR analysis using genomic DNA isolated from the mouse tail with the primers for control, TPST1 wild-type allele, TPST1 mutant allele, APOE wild-type allele, and APOE mutant allele. Product lengths of the control, TPST1 wild-type allele, TPST1 mutant allele, APOE wild-type allele, and APOE mutant allele were 200 bp, 724 bp, 601 bp, 155 bp, and 245 bp, respectively. A25 and A26 represented TPST1+/-/ApoE-/-.The genotypes of TPST2-/-/ApoE-/- mutant mice were determined by PCR analysis with the primers for control, TPST2 wild-type allele, TPST2 mutant allele, APOE wild-type allele, and APOE mutant allele. Product lengths of the control, TPST2 wild-type allele, TPST2 mutant allele, APOE wild-type allele, and APOE mutant allele were 200 bp, 484 bp, 805 bp, 155 bp, and 245 bp, respectively. A231, A232, and A237 represented TPST2+/-/ApoE-/-, while A233, A234, A235, and A236 represented TPST2+/+/ApoE-/-. M, 100 bp plus DNA ladder. WT, wild type; MUT, mutation; H20, negative control. TPST, tyrosylprotein sulfotransferases [file 12915_2023_1641_MOESM13_ESM.tif]
