# Supplementary material for: Enhanced tyrosine sulfation is associated with chronic kidney disease-related atherosclerosis
Source: BMC Biol. 2023 Jul 10;21:151. doi: 10.1186/s12915-023-01641-y (PMC10332009; doi:10.1186/s12915-023-01641-y)
Supplement: Supplementary file 9 — Additional file 9: Table S2. Genotype identification results for F0 mice. [file 12915_2023_1641_MOESM9_ESM.doc]

| Gene type | Sequencing（5’-3’） | Note |
| --- | --- | --- |
| Wild type | TTACTGTTAAAACGTTTTGTTCTTTCCATGGTGAGTCTTTCCATGGTGAGTCAGATGCATAGCTTCAGATTTGTTAAATAAAAAAATGCTGTTTCTGATT……CAATTGTGAGTGACCTGACTTCTGGTTTCTTTTTTCCCCTCCTTAAGTGTAAGGACCACTCTGCGTTTGAGGCTTTGTTTGGATGAGGTGTGGTTTCTTG |  |
| TPST1 knockout | TTACTGTTAAAACGTTTTGTTCTTTCCATGGTGAGTCTTTCCATGGT…(-13776bp)…TTAAGTGTAAGGACCACTCTGCGTTTGAGGCTTTGTTTGGATGAGGTGTGGTTTCTTG | -13776bp |
