# Supplementary material for: Enhanced tyrosine sulfation is associated with chronic kidney disease-related atherosclerosis
Source: BMC Biol. 2023 Jul 10;21:151. doi: 10.1186/s12915-023-01641-y (PMC10332009; doi:10.1186/s12915-023-01641-y)
Supplement: Supplementary file 10 — Additional file 10: Table S3. Primers for verification of vector construction. [file 12915_2023_1641_MOESM10_ESM.doc]

| Primer | Sequence (5’→3’) |
| --- | --- |
| P1 | GCTTAGGGAACGCATCAC |
| P2 | TCCACGCCGAGCTGTATTCATTTT |
| P3 | GGGAAATGGAGCCAGCAAGAT |
| P4 | TCACGACCATAAGAACAGAATA |
| P5 | CAGGCTGTGACGATTTTT |
| P6 | GAGGTGAGGGAAGCAGTTA |
| P7 | TGCCAGGATCAATACAGTT |
| P8 | AGAGCCTCAGGTGGACAGCAAGAT |
| P9 | CAAATGTTGCTTGTCTGGTG |
| P10 | GTCAGTCGAGTGCACAGTTT |
| P11 | GCCTAGCCGAGGGAGAGCCG |
| P12 | TGTGACTTGGGAGCTCTGCAGC |
| P13 | GCCGCCCCGACTGCATCT |
